# Quantitative profiling of glycerophospholipids during mouse and human macrophage differentiation using targeted mass spectrometry

**DOI:** 10.1038/s41598-017-00341-2

**Published:** 2017-03-24

**Authors:** Cuiping Zhang, Yi Wang, Fang Wang, Zhenxin Wang, Yu Lu, Ying Xu, Ke Wang, Huali Shen, Pengyuan Yang, Shan Li, Xue Qin, Hongxiu Yu

**Affiliations:** 1 Department of Clinical Laboratory, First Affiliated Hospital of Guangxi Medical University, Nanning, 530021 Guangxi China; 2 Department of Systems Biology for Medicine and Institutes of Biomedical Sciences, Shanghai Medical College, Fudan University, Shanghai, 200032 China

## Abstract

Macrophage lipid metabolism plays a pivotal role in innate and adaptive immune responses. Previous studies have shown that this process plays a role in infections and contributes to the pathogenesis of diabetes, atherosclerosis, and other immunometabolic diseases. M1 macrophages, or classically activated macrophages, are key players in the defense against bacterial infections. M2 macrophages, or alternatively activated macrophages, are involved in anti-inflammatory responses. Using the multiple reaction monitoring method, we identified changes in lipid composition during the differentiation of human and murine macrophages. We detected over 300 lipid molecules in mammalian macrophages, and we observed a striking shift in the composition of glycerophospholipids (GLs) from saturated and monounsaturated to polyunsaturated during human macrophage polarization. Moreover, M2 macrophages showed a higher level of lysophospholipids (lysoGLs) than did M1 macrophages. The lysoPI species increased in human and mouse M2 macrophages, suggesting that they may be involved in M2 macrophage polarization and anti-inflammatory processes. Collectively, these results indicate that lipids may play a role in the pro- and anti-inflammatory activities of macrophages and may be markers of the macrophage activation state.

## Introduction

Macrophages, nonspecific components of the innate immune system, are present in all tissues of the body. When a monocyte is attracted to a damage site by chemokines, it undergoes a series of changes and transforms into a macrophage. Macrophages participate in numerous biological processes, ranging from sensing cell injury and infection to bone regeneration and wound healing^1^. They also contribute to the pathogenesis of several immunometabolic diseases, including diabetes, obesity, and atherosclerosis^1–3^.

Depending on the activation stimuli, macrophages are classified into two main polarization groups: classically activated M1 macrophages (pro-inflammatory phenotype) and alternatively activated M2 macrophages (anti-inflammatory phenotype)^4^. M1 macrophages can be activated by external stimuli, such as the Toll-like receptor TLR4 agonist lipopolysaccharide (LPS) from *E. coli*, interferon-gamma (IFN-γ), tumor necrosis factor-alpha (TNF-α), and granulocyte-macrophage colony stimulating factor (GM-CSF) and by the production of interleukin-6 (IL-6) and interleukin-12 (IL-12)^1^. M1 macrophages are potent effector cells that provide the first line of defense against infections, and they respond to infections within hours to days. In contrast, M2 macrophages can be induced by interleukin-4 (IL-4) and interleukin-13 (IL-13), and they produce anti-inflammatory factors, such as transforming growth factor-beta (TGF-β) and interleukin-10 (IL-10) to resolve inflammations and promote tissue repair and remodeling^3, 5^. The proper polarization and functioning of M1 and M2 macrophages require the reprogramming of intracellular metabolism, where by M1 macrophages increase aerobic glycolysis and reduce mitochondrial respiration. Pro-inflammatory metabolism is essential for producing precursor molecules, such as lipids and amino acids^5^. It also sustains the generation of inflammatory factors^5^. By contrast, M2 macrophages tend to use oxidative metabolic processes, ranging from fatty acid uptake and oxidation to oxidative phosphorylation and mitochondrial respiration, to perform their long-term functions^6, 7^. Fatty acid β-oxidation is anti-inflammatory, probably because of the decrease in the production of prostaglandin^8^.

Lipid metabolism may contribute to the pro- or anti-inflammatory functions of macrophages by meeting energetic requirements and modulating membrane fluidity^9^. Lipids, such as free fatty acids (FFAs), neutral lipids, glycerophospholipids (GLs), and sphingolipids, are components of the cell membrane and lipoproteins; they are also involved in multiple biological processes, such as inflammation and cell differentiation^10, 11^. With the development of mass spectrometric approaches, the analysis of global lipids has provided new insights into the characterization of the alteration of lipids associated with diseases and has provided a better understanding of their pathophysiology^12^. Recent studies have revealed a network of interactions between metabolic profiles and transcription data to explore the modulatory mechanisms of immune cell differentiation^6, 13, 14^.

However, few studies have focused on lipid metabolism patterns in the complete processes of monocyte-macrophage differentiation and macrophage polarization. The present study investigated the dynamic lipid profiles associated with different stages of macrophages. We identified more than 300 lipid molecules in mammalian macrophages using liquid chromatography-mass spectrometry (LC-MS) with multiple reaction monitoring (MRM) scanning, which simultaneously detects a broad spectrum of lipid molecules. The main lipid classes identified were phosphatidylcholine (PC), phosphatidylserine (PS), phosphatidylethanolamine (PE), phosphatidylinositol (PI), phosphatidylglycerol (PG), sphingomyelin (SM), and ceramide (Cer). We observed a striking shift in the composition of the GLs from short-chain saturated and monounsaturated lipids to long-chain polyunsaturated lipids in activated human macrophages derived from THP-1 cells. Mouse M2 macrophages had higher levels of lysoGLs than did mouse M1 macrophages. In addition, during the polarization of human and mouse macrophages, we noticed that the lysoPI level was significantly increased in IL-4-induced M2 macrophages. Our results describe a dynamic lipid profile during monocyte-to-macrophage differentiation and macrophage polarization. This distinct lipid molecular analysis can shed new light on monocyte-to-macrophage differentiation and macrophage polarization and help uncover new candidate markers of the inflammatory response.

## Results

### Quantification of the lipid profiles of mammalian macrophages

**Figure 1:**
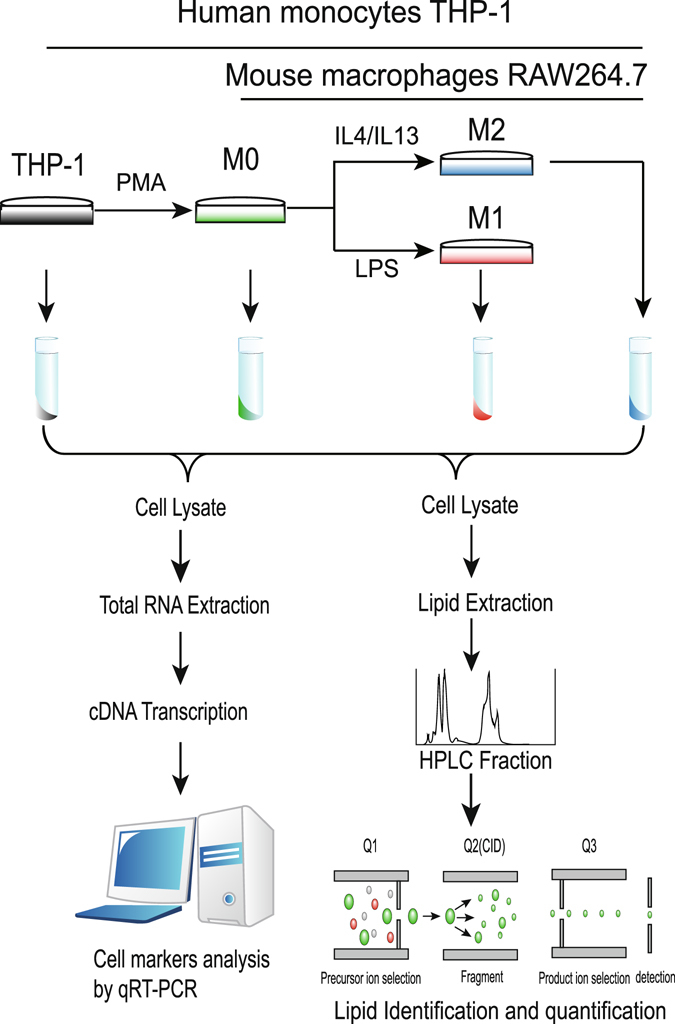
Schematic diagram of macrophage polarization and lipid detection.

To investigate the lipid profiles during macrophage differentiation, we developed an MRM-MS method. Using this method, we detected nearly 500 lipid molecules, including five classes of GLs and three classes of sphingolipids. Supplementary Table 1 lists the information about the lipid molecules that can be detected using our MRM-MS method. In human monocyte THP-1 cells, 368 lipid molecules were detected (250 and 216 in the positive and negative ion modes, respectively, with 98 overlapping between the two modes). In the mouse macrophage RAW264.7 cell line, we identified 420 lipid molecules (240 and 273 in the positive and negative ion modes, respectively, with 93 overlapping between the two modes). Most lipids were GLs, and a fraction was sphingolipids. We performed the experiments three times. In each replicate, 10^7^ cells (either human or mouse cells) were used to extract the RNA and lipids and to perform the MRM-MS analysis. Figure 1 shows the workflow of the entire experiment.

### Activated macrophages derived from human monocytic THP-1 cells show the accumulation of medium- and long-chain polyunsaturated lipids

**Figure 2:**
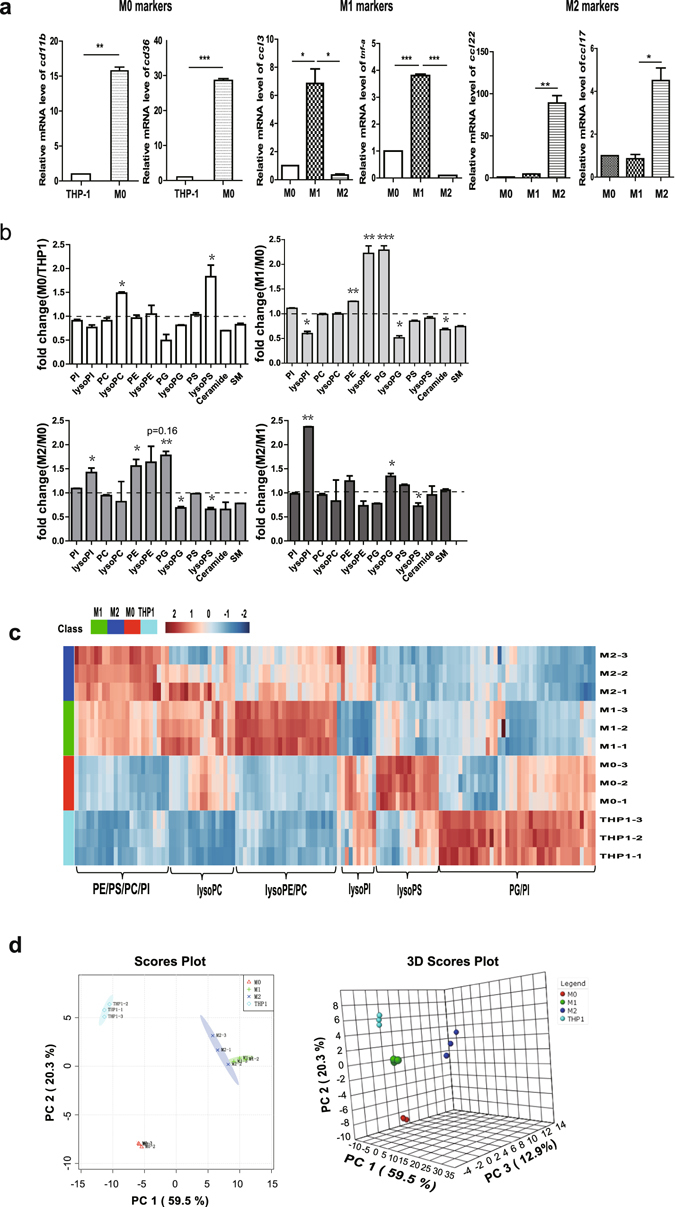
Activated macrophages derived from human monocytic THP-1 cells show an increase in lysoPE, PE, and PG and a decrease in lysoPG. (**a**) Left to right, *cd11b* and *cd36* were analyzed to identify the differentiation of inactivated macrophages. The expression levels of *ccl3*, *tnf-a*, *ccl22*, and *ccl17* were analyzed to assess the polarization into M1 and M2 macrophages. The data represent the mean ± SEM, n = 3, *p < 0.05, **p < 0.01, ***p < 0.001. (**b,c**) Lipids were extracted from macrophages in different differentiation stages and then identified using the MRM method. The lipid classification between two groups was compared using Student’st-test. Histogram and clustering analysis demonstrated increased lysoPC and lysoPS in inactivated macrophages compared to THP-1 monocytes. Activated macrophages, including M1 and M2 cells, showed increased levels of PE, lysoPE, and PG and downregulation of lysoPG. A comparison of M1 and M2 cells indicated that the lipid classification of lysoPI was dramatically increased in M2, whereas that of lysoPS was increased in M1 cells. (**d**) PCA indicated that four groups of cells could be separated from each other in a 2D score plot (up) and further divided in a 3D score plot (below).

**Figure 3:**
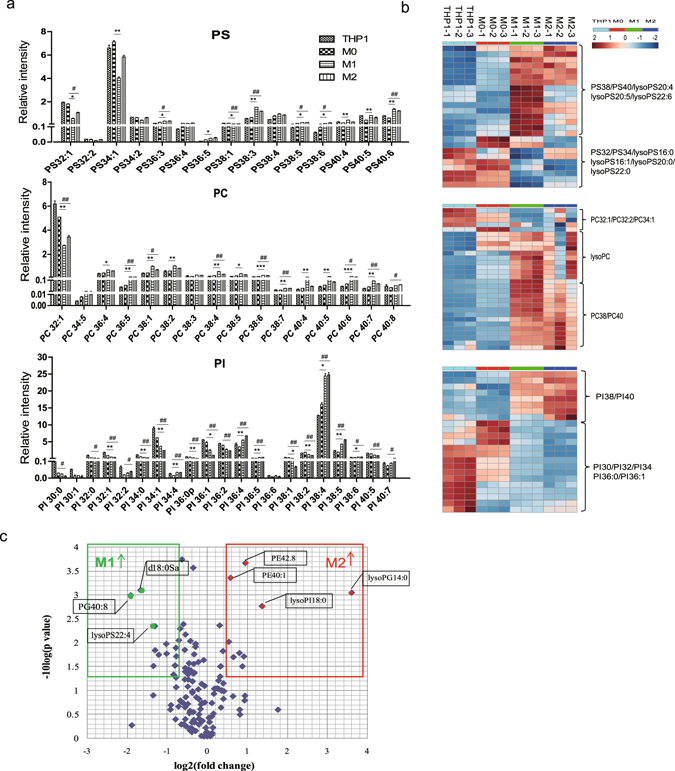
Activated macrophages derived from human monocytic THP-1 cells show the accumulation of medium- and long-chain polyunsaturated lipids (**a,b**) Histogram (a) and clustering analysis (b) indicated that during macrophage differentiation, long-chain and polyunsaturated lipids (PS38:3, PS38:5, PS40:6, PC40:6, and PI38:4, etc.) accumulated in the activated macrophages. In contrast, saturated and monounsaturated lipids with fewer carbons (PS32:1, PC32:1, PI30:0, PI34:1, and PI36:1, etc.) were elevated in THP-1 and M0 cells (* represents comparisons between M1 and M0, # represents comparisons between M2 and M0). (**c**) Volcano plots show that lipids were differentially expressed between M1 and M2 macrophages. The x-axis shows the log2-fold change (fold change = intensity of M2 to M1). The y-axis shows the -log10 p-value for the corresponding lipids. The top lipids that are elevated in M1 cells are highlighted in green, and those that are elevated in M2 cells are indicated by red dots (fold change >1.5 or < 0.67, *p* < 0.05).

During PMA-induced monocytic THP-1 cell differentiation, the intensities of 139 lipid species changed significantly (fold change >1.5 or <0.67, *p* < 0.05). Human monocyte THP-1 cells were differentiated into macrophages with phorbol-12-myristate-13-acetate (PMA) and then polarized into M1 and M2 macrophages. The gene expression of *cd36* and *cd11b* was analyzed to identify inactivated macrophages (uM0). The polarization into M1 and M2 macrophages was assessed using the expression levels of *ccl3*, *tnfα*,*ccl22*, and *ccl17*, as assayed using quantitative reverse transcription polymerase chain reaction (qRT-PCR) (Fig. 2a). M1 and M2 cells were used as activated macrophages^15^. Univariate analysis showed that during the differentiation of monocytes to macrophages, the lysoPC and lysoPS levels increased, and the PG level decreased. The intensity of lysoPC and lysoPS increased more than 1.5 times in uM0 cells compared with THP-1 cells; however, the PG intensity decreased by 50% without a statistically significant difference (Fig. 2b). We further investigated the lipid compositions of M1 and M2 macrophages. PE and PG were significantly upregulated in M1 cells, whereas lysoPG was downregulated in both activated macrophages compared with the uM0 group. The lysoPI level increased dramatically; however, the lysoPS level decreased in the M2 cells compared with that in the M1 cells (Fig. 2b). Multivariate analysis results, such as those of clustering analysis, were consistent with these results (Fig. 2c). Principal component analysis (PCA) indicated that four groups of cells could be separated from each other in a two-dimensional (2D) score plot and could be further separated in a three-dimensional (3D) score plot (Fig. 2d). The lipid peaks responsible for separating the different cell populations by PCA are shown in the corresponding loading plot (Supplementary Fig. 6a). In the next step, we analyzed the variation of each type of lipid molecule during macrophage differentiation. We detected a striking shift in the GL composition from saturated and monounsaturated lipids to polyunsaturated lipids, such as PS, PC, and PI. Specifically, the inactivated cells e.g., THP-1 and uM0 macrophages, showed higher levels of saturated and monounsaturated lipids and lower levels of polyunsaturated lipids, whereas the activated macrophages e.g., M1 and M2 macrophages, showed lower levels of saturated and monounsaturated lipids and higher levels of polyunsaturated lipids (Fig. 3a,b). Moreover, the lipid molecules were elongated in the activated macrophages (Fig. 3a,b). M2 macrophages had higher lysoPG14:0 levels than did M1 macrophages (>10-fold change). PG40:8, lysoPS22:4, and d18:0 sphingosine were higher in M1 macrophages than in M2 macrophages (>2-fold change, Fig. 3c). In summary, the PE, lysoPE, and PG levels increased in the human activated macrophages. The M2 macrophages showed a high level of lysoPI and a low level of lysoPS. Depth analysis of the variation of each lipid species during macrophage polarization showed an accumulation of medium- and long-chain polyunsaturated lipids in the activated macrophages.

To confirm the lipidomic characteristics observed in the THP-1 cells, we performed experiments using human peripheral blood mononuclear cells (PBMCs). Using these methods, we isolated human primary monocytes and performed lipid extractions and MRM analysis. During human primary monocyte-macrophage differentiation, PC, PS, and PA species showed a decrease in polyunsaturated fatty acids (PUFA) accompanied by an increase in monounsaturated fatty acids (MUFA) and saturated species (Supplementary Fig. 2b), results that agree with previously reported data^16^. The lysoPC, lysoPS, and lysoPI intensities increased significantly in M-CSF–derived macrophages compared with human monocytes, which agrees with the results for THP-1 cells (Supplementary Fig. 2a and b). Additionally, an increase in PC, PE, and PI species was found in the LPS-induced M1 macrophages (Supplementary Fig. 2c). M2 cells showed the opposite tendency, with a decrease in PC, PE, and PI species and an increase in PG species (Supplementary Fig. 2d).

These results indicated that the lipidomics profiles of THP-1 cells and human primary monocyte-derived macrophages were consistent, although each has some level of specificity. During monocyte-macrophage differentiation, THP-1 cells and human primary monocytes showed an increase in lysoPC and lysoPS but a decrease in PUFA, while human primary monocytes also had an increase in MUFA and saturated species. During macrophage polarization, THP-1 cells showed an increase in PE and PG species and a shift from MUFA to PUFA accompanied by elongated fatty acids, whereas human primary monocytes showed a decrease in lysoGLs. Supplementary Table 3 shows a comparison of the lipidomic results from THP-1 cells and human PBMCs.

### Mouse M2 macrophages have higher levels of lysophospholipids than do M1 macrophages

**Figure 4:**
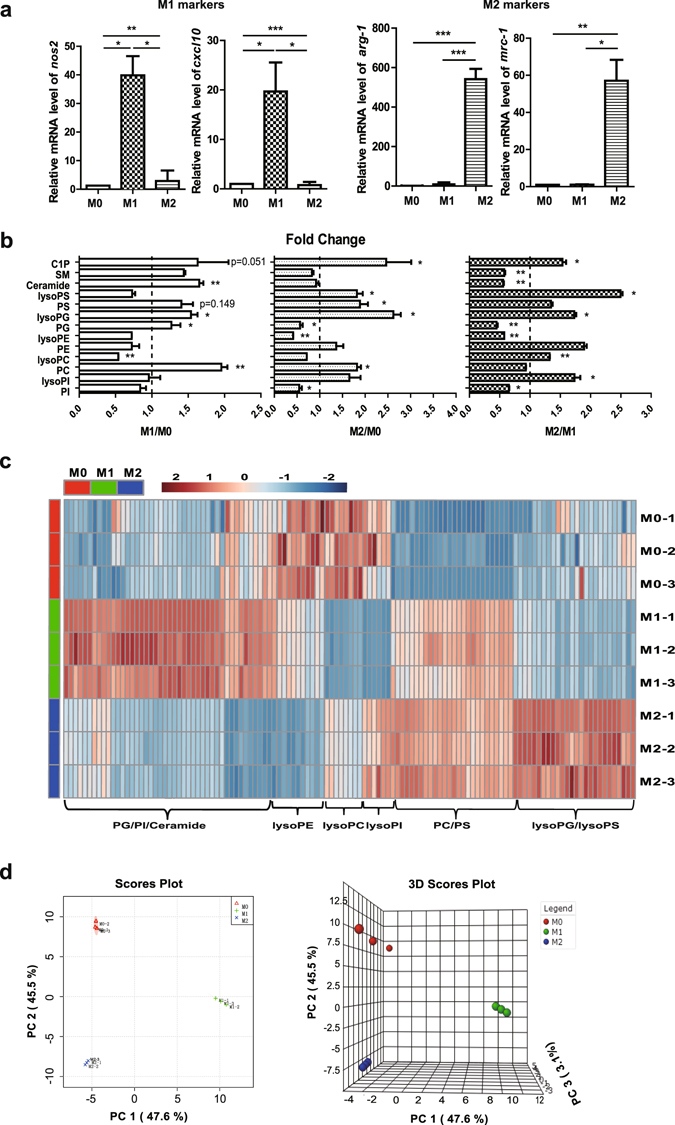
Most glycerophospholipids and lysophospholipids are increased in mouse activated macrophages. (**a**) From left to right, the expression levels of *nos2*, *cxcl10*, *arg-1*, and *mrc-1*were analyzed to assess the polarization into M1 and M2 macrophages. The data represent the mean ± SEM, n = 3, *p < 0.05, **p < 0.01, ***p < 0.001. (**b,c**) Histogram and clustering analysis show a comparison of the lipid classification between the two groups. Most glycerophospholipids and lysophospholipids were elevated in activated macrophages. Ceramide and ceramide-1-phosphate were also increased in activated cells. (**d**) PCA suggested that three types of macrophages could be separated from each other in 2D and 3D score plots.

**Figure 5:**
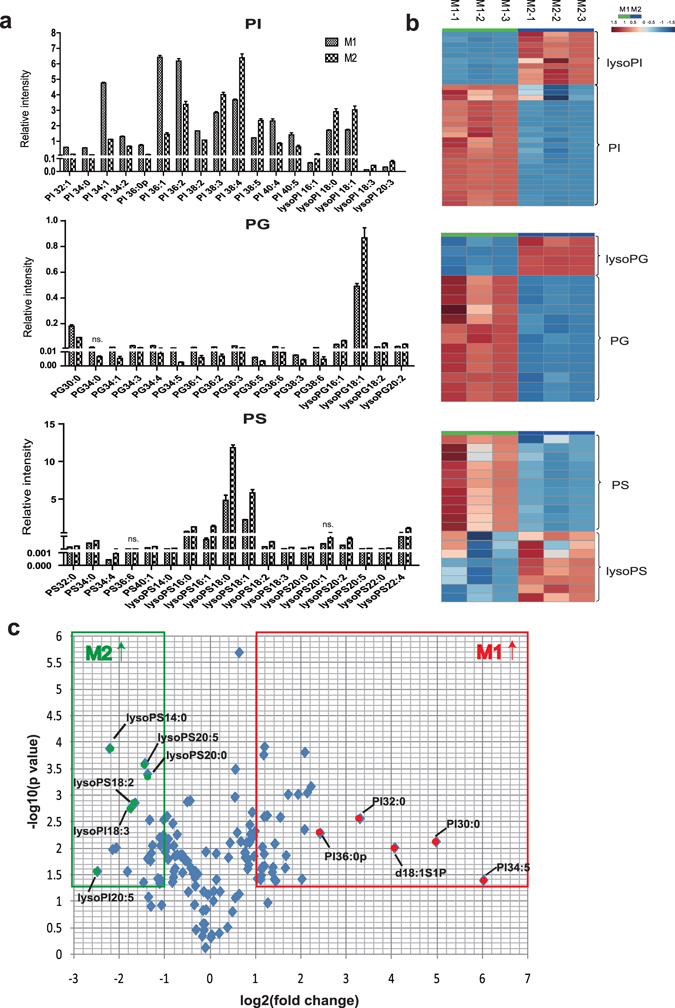
Mouse M2 macrophages have higher levels of lysophospholipids than do M1 macrophages. (**a,b**) As shown in the histogram and heatmap, lysophospholipids, including lysoPI, lysoPG, and lysoPS, were increased in M2 macrophages, whereas PI, PG, and PS were increased in M1 macrophages. (**c**) Volcano plots show that lipids were differentially expressed between M1 and M2 macrophages. The x-axis shows the log2-fold change (fold change = intensity of M1 to M2). The y-axis shows the-log10 p-value for the corresponding lipids. The top lipids that are elevated in M1 cells are highlighted in red, and those that are elevated in M2 cells are indicated by green dots (fold change >1.5 or <0.67, *p* < 0.05).

Inactive macrophages (uM0) from the mouse macrophage RAW264.7 cell line were treated with LPS, the bacterial molecule that specifically activates TLR4 and induces an inflammatory response in M1 macrophages. In addition, IL-4 stimulated the quiescent macrophages to polarize into M2 cells (Fig. 1). We analyzed specific markers using qRT-PCR to evaluate the polarization of the cells. The expression of the M1 markers NOS2 and CXCL10 was elevated significantly in the macrophages treated with LPS. The macrophages treated with IL-4 expressed a high level of the M2 markers Arg-1 and Mrc-1 (Fig. 4a). To study the macrophage lipid profile in response to different inflammatory stimuli, RAW264.7 cells were also subjected to lipid extraction and MRM-MS analysis, just as the THP-1 cells had been. In the RAW264.7 cells, 126 differential lipids were identified. LPS treatment of RAW264.7 cells resulted in elevated levels of most sphingolipids, including SM and Cer, as well as their phosphates. In M2 macrophages, a similar increase was observed for ceramide-1-phosphate (C1P) but not for SM or Cer (Fig. 4b). Significant changes were observed in the GL and lysoGL levels after LPS and IL-4 stimulation, respectively. The PS, lysoPG, PG, and PC levels increased and the lysoPC level decreased in M1 cells relative to uM0 cells. The M2 macrophages showed an increase in the GL and lysoGLs level (PS, PE, PC, lysoPS, lysoPG, and lysoPI) relative to the uM0 cells. The PG,PI, and lysoPE levels were significantly decreased in M2 cells (Fig. 4b,c). The bargraph and heatmap clearly indicate the increasing trend of lysoGL levels in response to IL-4 stimulation (Fig. 4b,c). PCA showed that the three groups of macrophages were separated by 126 differentially expressed lipids (Fig. 4d). Supplementary Fig. 6b shows the corresponding loading plot. We observed that the PI, PG, and PS levels increased in the M1 macrophages, whereas their corresponding lysoGL forms increased significantly in the M2 macrophages (Fig. 5a,b). According to the volcano plot, the lipids that were most elevated in the M1 macrophages were the PI species and the sphingosine-1-phosphate lipids (>5-fold change). The lysoPS species specifically increased in M2 cells (>2.5-fold change), as shown in Fig. 5c. Partial least square regression discriminant analysis (PLS-DA) was performed, as shown in Supplementary Fig. 1. The uM0 cells and activated macrophages were separated from each other using PLS-DA. Furthermore, lipids with variable importance in projection (VIP) ≥1 were selected as potential markers and confirmed by t-test (Table 1). PG40:8 was the lipid that played the greatest role in differentiating human M1macrophages from THP-1 and M2 cells. Specifically, the levels of saturated and monounsaturated PIs, such as PI36:1 and PI36:0, decreased in human M2 cells, which contributed to dividing M2 cells from uM0 cells. In mouse macrophages, lysoPE and lysoPC played the greatest role indistinguishing between M2 and uM0 cells. Supplementary Table 3 shows a comparison of the lipidomics between human and mouse macrophages.

**Table 1:** Lipid markers for distinguishing macrophages in different stages.

Group A vs. Group B	Lipid	VIP	Intensity of group A (mean ± SD)	Intensity of group B (mean ± SD)	*p*-value
HumanTHP1 vs. uM0	PS40:5 (18:0/22:5)	1.141	8.10E-01 ± 1.29E-03	4.30E-01 ± 3.43E-03	0.000
	PE32:1 (14:0/18:1) or (16:0/16:1)	1.141	1.62E + 00 ± 1.60E-04	1.12E + 00 ± 3.19E-03	0.000
	C1P12:0	1.141	3.43E-03 ± 1.68E-04	7.07E-04 ± 3.29E-06	0.002
Human M1 vs. THP1	PG40:8	1.067	7.77E-04 ± 2.50E-05	1.82E-04 ± 4.19E-07	0.001
	lysoPS20:5 (sn-1)	1.067	3.93E-02 ± 1.68E-03	5.06E-03 ± 2.55E-04	0.001
	SM18:0	1.067	2.55E + 00 ± 2.81E-02	5.90E + 00 ± 1.16E-01	0.001
Human M2 vs. THP1	PI 36:1 (18:0/18:1)	1.127	1.01E + 00 ± 2.30E-02	5.62E + 00 ± 3.29E-02	0.000
	PI 36:0p (16:0p/22:6)	1.127	1.04E-01 ± 6.26E-03	6.05E-01 ± 1.64E-02	0.001
	PS38:4 (18:0/20:4)	1.127	8.68E-01 ± 2.64E-02	4.86E-01 ± 6.74E-03	0.003
Human M1 vs. uM0	PG34:1 (16:0/18:1)	1.064	1.81E + 00 ± 1.33E-02	5.64E-01 ± 8.02E-03	0.000
	PS40:5 (18:0/22:5)	1.062	8.17E-01 ± 8.31E-03	4.30E-01 ± 2.43E-03	0.000
	PS40:4 (18:0/22:4) or (20:1/20:3)	1.062	4.29E-01 ± 5.41E-03	1.99E-01 ± 2.19E-03	0.000
Human M2 vs. uM0	PI 36:0p (16:0p/22:6)	1.188	1.04E-01 ± 6.26E-03	4.94E-01 ± 1.36E-02	0.000
	lysoPS22:0 (sn-1)	1.188	2.73E-02 ± 5.72E-03	8.00E-02 ± 1.55E-05	0.005
	PI 36:1 (18:0/18:1)	1.187	1.01E + 00 ± 2.30E-02	4.81E + 00 ± 2.71E-01	0.002
Human M1 vs. M2	PG40:8 (20:4/20:4)	1.203	7.77E-04 ± 2.50E-05	2.06E-04 ± 6.07E-06	0.001
	PC40:7 (18:1/22:6)	1.202	9.40E-02 ± 2.19E-03	7.01E-02 ± 2.42E-03	0.009
	PE42:8 (20:2/22:6)	1.202	5.95E-03 ± 8.47E-05	1.15E-02 ± 7.70E-05	0.000
Mouse M1 vs. M0	PI 32:0 (16:0/16:0)	1.122	2.60E-01 ± 1.22E-02	1.50E-02 ± 1.20E-03	0.000
	PC32:0 (16:0/16:0)	1.121	1.75E + 00 ± 4.48E-02	7.25E-01 ± 4.11E-02	0.003
	PC40:6 (18:0/22:6)	1.121	1.25E-01 ± 1.78E-03	6.58E-02 ± 1.31E-03	0.001
Mouse M2 vs. M0	lysoPE 18:1 (sn-1)	1.170	1.63E + 00 ± 3.27E-02	4.24E + 00 ± 4.46E-02	0.000
	lysoPC 20:1 (sn-1)	1.169	1.09E-01 ± 3.78E-03	2.01E-01 ± 3.51E-03	0.003
	lysoPE 20:4 (sn-2)	1.169	1.19E + 00 ± 5.34E-02	2.64E + 00 ± 2.47E-02	0.002
Mouse M1 vs. M2	PI 34:0 (16:0/18:0)	1.059	5.68E-01 ± 1.10E-02	1.22E-01 ± 4.11E-03	0.001
	PI 38:3 (18:0/20:3) or (18:1/20:2)	1.059	2.84E + 00 ± 7.45E-02	4.02E + 00 ± 1.39E-01	0.017
	lysoPE 22:6 (sn-1)	1.059	1.42E + 00 ± 2.97E-02	6.23E-01 ± 2.74E-02	0.003

These results demonstrate that the activation of mouse macrophages by inflammatory mediators altered most GL categories, and mouse M2 macrophages showed a higher level of lysoGLs than did M1 macrophages. Additionally, we noted that the lysoPI levels increased significantly in IL-4-induced M2 macrophages.

## Discussion

The immune system continually senses and responds to environmental stimulations, which requires a substantial amount of biological energy. Because immune cells lack significant stores of nutrients, they dramatically increase their uptake of glucose, amino acids, and fatty acids from their microenvironment when responding to external threats^17^. Thus, metabolic reprogramming is a critical step in the maturation and polarization of immune cells^17^.

M1 macrophages induced by LPS and IFN-γ prefer to use glycolysis^14^. IL-4-activated M2 macrophages mainly employ the metabolic pathways of aerobic glycolysis metabolic and of fatty acid oxidation^5, 14^. Lipid metabolism significantly contributes to the plasticity of human and mouse activated macrophages based on transcriptomic, metabolomic, and lipidomicanalyses^6, 13, 18–20^. Lipidomic studies^18, 19^ on activated macrophages have been performed using non-target ultra-performance liquid chromatography-mass spectrometry (UPLC-MS); however, these studies only focused on one step of the macrophage differentiation process. Few studies have focused on the large-scale, dynamic lipidomic observation of macrophage differentiation and polarization. In the current study, we developed a method, based on MRM-MS, that enables the parallel, targeted identification of nearly 500 lipid molecules. MRM measurement has been applied to the metabolomic analysis and detection of the post-translational modifications of proteins, with high sensitivity and quantitative accuracy^21, 22^. We used this approach to identify a set of lipids that defines the M1 and M2 stages of macrophage polarization. In human THP-1 cells, we detected a total of 368 lipids and found 139 differentially expressed lipids. In murine RAW264.7 macrophages, we detected a total of 420 lipids and 126 differentially expressed lipids.

Based on the transcriptomic and lipidomic profiles during human monocyte-macrophage differentiation, Ecker *et al*. revealed that the major lipid class switches from cholesterol in monocytes to PC in macrophages^16^. Wallner *et al*. demonstrated that PE plasmalogens (C16 and C18) showed a decrease in PUFA and an increase in MUFA during the differentiation of human primary monocytes and HL-60 cell macrophages^23^. In our experiments, we did not observe any significant changes during monocyte-macrophage differentiation when using the THP-1 cell line. Unexpectedly, we found that GLs in MUFA and the saturated species decreased and that GLs in the long-chain PUFA increased during THP-1-derived macrophage polarization (Fig. 3). This change was probably due to the massive upregulation of fatty acid elongase and desaturase when immune cells differentiate, according to the transcriptome information reported by Ecker *et al*.^16^ and Wallner *et al.* ^24^. Moreover, the corresponding lipidomic study of GM-CSF/IL-4-dependent dendritic cell (DC) differentiation was considered^25^. Josef Ecker and colleagues demonstrated that GM-CSF/IL-4-differentiated monocytes, compared with M-CSF-differentiated monocytes, have fewer MUFA and phospholipid species, which is due to the lower stearoyl-CoA desaturase activity observed in DCs^25^.

PMA-differentiated THP-1 cells with multiple cytogenetic alterations, which differ from growth-arrested macrophages, have some limitations for lipidomic analyses. To confirm the results observed in the THP-1 cells, we designed an experiment to characterize the lipidomic profile of human monocytes undergoing differentiation into mature macrophages in the presence of M-CSF and their subsequent polarized activation into M1 or M2 cells. The cell culture process used was the same as that used in previous studies^20, 23, 24^. The use of serum during macrophage differentiation may affect lipidomic analyses because serum contains multiple regulatory lipid speciesthat may cause FBS-batch-dependent variable responses^24, 26^. Thus, we used the same batch of serum to avoid artificial effects. We detected the lipidomic profiles of human PBMCs and found a decrease in PUFA accompanied by an increase in MUFA and the saturated species, especially PC, PS, and PA species, during human primary monocyte-macrophage differentiation. These results are in agreement with a previous report (Supplementary Fig. 2b)^27^.

In our study, we also observed the lipid profiles of human M1 and M2 macrophages derived from THP-1 cells or human PBMCs. Ruipérez *et al*. demonstrated the generation of lysoPC upon LPS stimulation by the phospholipase hydrolysis of phospholipids^28^. Julio *et al*. analyzed lipid changes in IL-4-treated macrophages and found that lysoPE is a phospholipase A2-induced product that is derived from PE membranes and that it may be involved in regulating phagocytosis^29^. In our study, changes in the lysoGL molecular species were observed during monocyte-macrophage differentiation and macrophage polarization (Supplementary Figs 2 and 3). Specifically, the findings were as follows: (1) In resting macrophages, the intensity of lysoPC, lysoPS, and lysoPI and of mono- and polyunsaturated lysoPC species was found to be significantly elevated (Supplementary Figs 2a and 3a). (2) An increase in the levels of PC, PE, and PI species was found in the LPS-induced M1 macrophages derived from human primary monocytes (Supplementary Fig. 2c), and the levels of long-chain polyunsaturated lipids were found to increase in THP-1 cell-derived M1 macrophages, especially in the lysoPC, lysoPS, and lysoPE species (Supplementary Fig. 3b). (3) Compared with M1 cells, M2 cells had decreased levels of PC, PE, and PI species and increased levels of PG species (Supplementary Fig. 2d). The lysoPG14:0 level in M2 macrophages was 12 times greater than that in M1 macrophages (Fig. 3c and Supplementary Fig. 3b). LysoPG has been shown to be a mediator of inflammation that can inhibit chemokine-induced migration and IL-1β production in human monocytes and neutrophils^30^. The lysoPI18:0 content is relatively high in M2 cells (>2.5-fold greater than that in M1 cells).

Arachidonic acid (AA), a precursor of the bioactive lipid mediator eicosanoid, incorporates into cellular phospholipids and forms AA-containing species (GLs with C20:4). The number of AA-containing species increases under cellular stimulation, and this increase is thought to compensate for the exhaustion of AA^31^. David *et al*. found that the levels of two AA-containing species, PI (20:4/20:4) and PC (20:4/20:4), increased in human peripheral blood monocytes treated with PMA^32^. In contrast, we did not observe significant changes in PI (20:4/20:4) or PC (20:4/20:4) in THP-1 cells treated with PMA. Nevertheless, many other AA-containing GLs were elevated in the PMA-induced macrophages (Supplementary Fig. 4a). Additionally, the levels of almost all AA-containing species we detected were increased in M1 and M2 macrophages in comparison to those in uM0 cells (Supplementary Fig. 4b). The VIP score and volcano plot showed that PG40:8 (C20:4/20:4) was highest in M1 macrophages compared with uM0 and M2 cells (Fig. 3c, Table 1, and Supplementary Fig. 4c). The elevation of AA-containing species suggests that they may be considered lipid biomarkers of the activation state of human macrophages.

Using mass spectrometry analyses, we obtained a quantitative lipid profile of the mouse macrophages that responded to the Kdo2-lipid A (KLA) treatment, a TLR4 agonist, thus demonstrating the synthesis of FFA and variations in glycerolipids, GLs, sphingolipids, and prenols^18, 19^. Most GL species, including PC, PE, PS, and PG, increased after 24 h of KLA treatment. Almost every category of sphingolipid that was analyzed, including sphingoid bases and their phosphate forms, sphingosine-1-phosphate (S1P), Cers, and C1P, were observed to increase in the KLA-stimulated macrophages^19^. The levels of Cers, SMs, S1P, and glucosylceramides increased upon TLR stimulation and modulated the TLR-induced release of IL-6^13^. We built a model of RAW264.7 macrophages that responded to LPS to mimic a bacterial infection. We found that most GLs increased (Fig. 4); moreover, our lipidomic data agreed with those of previous studies^19^. The PI species of lipids were highest in the M1 macrophages compared to the M2 macrophages (Table 1 and Fig. 5c). However, the levels of the PI species that contained C20:4, such as PI (18:0/20:40) and PI (20:4/20:4), did not increase significantly in the M1 macrophages, perhaps because LPS alone is a poor trigger for C20:4 generation, as shown in previous studies^33^.

Compared with the M1 macrophages, we observed that the levels of lysoGLs, especially the lysoPS and lysoPI species, increased significantly in the mouse M2 macrophages (Fig. 5). As a lipid mediator, lysoPS interacts with specific receptors to exert an effect on biological processes^34^. On one hand, lysoPS serves as an endogenous anti-inflammatory mediator that recruits macrophages to scavenge neutrophils, and it plays a key role in resolving inflammation^35–37^. On the other hand, lysoPS has been suggested to play a role in neuroinflammation^38^. Mice with a deficiency of G protein coupled receptor 34, the lysoPS receptor, showed less immune cell infiltration and higher levels of cytokine production upon bacterial infection than did wild-type mice^39^. These observations suggest that lysoPS acts as a lipid mediator of inflammation, and it may play a pro-inflammatory role^39^. The *in vivo* effect of lysoPS is unclear, and further studies are needed to explain its biological significance.

As a result of evolution, there are many differences between rodent and human macrophages^40, 41^. The use of the mouse macrophage RAW264.7 cell line has a limitation that would affect the glycerophospholipid profile due to the proliferative response. There are limitations of each model for lipidomic analysis. A comparison of the lipidomic profiles of mouse and human macrophages is shown in Supplementary Table 3. Our dynamic observations indicated that the lipid variations in human and mouse activated macrophages shared a similar characteristic, namely, that the levels of the lysoPI species increased in the M2 macrophages relative to those in the M1 macrophages (Figs 2b and 4b). This observation suggests that lysoPI may be involved in M2 macrophage polarization and anti-inflammatory processes. LysoPI is a second messenger that regulates a wide range of physiological processes, such as inflammation, angiogenesis, and atherosclerosis^34, 42, 43^. When coordinated with its receptor, lysoPI leads to neutrophil polarization and the abrogation of reactive oxygen species (ROS) formation, thus limiting inflammation^44^. Additionally, our results suggest that the human and mouse macrophages showed different lipid profile features. In human macrophages, medium- and long-chain polyunsaturated lipids accumulated in the activated cells. In mouse macrophages, the PI, PG, and PS levels increased in M1 macrophages, whereas their corresponding lysoGL forms significantly increased in M2 macrophages. Collectively, the differential levels of lipids detected in this study could help identify macrophage stages and serve as a therapeutic target to modulate the inflammatory state.

## Methods

### Reagents and materials

PMA,LPS, and formic acid were purchased from Sigma-Aldrich. Mouse IL-4, human IL-4, and human IL-13 were obtained from PeproTech. High-performance liquid chromatography (HPLC)-grade chloroform, methanol, isopropanol (IPA), and hexane were used without further purification. Phosphoricacid, potassium chloride, and ammonium acetate were obtained from Sinopharm Chemical Reagent Co., Ltd. The water was purified using a 0.22-µm Milli-Qfilter (Millipore, USA).

### Cell cultures

Mouse macrophage RAW264.7 cells and human monocyte THP-1 cells were maintained according to the American Type Culture Collection (Manassas, VA) guidelines. Both cell types were cultured with 10% heat-inactivated serum without the addition of CSF. The THP-1 cells were cultured on Roswell Park Memorial Institute-1640 (RPMI-1640) media containing 200 nmol/L PMA for 24 h and were allowed to differentiate into uM0 cells. Then, the uM0 cells were further induced into M1 macrophages using 100 ng/ml LPS and into M2 macrophages using 20 ng/ml IL-4 and 20 ng/ml IL-13. A total of 10^7^ cells were harvested, and the total RNA and lipids were isolated after 72 h of stimulation. Similarly, the RAW264.7 cells were stimulated with 100 ng/ml LPS or 20 ng/ml murine IL-4 for M1 or M2 cell polarization, respectively. After 12 h, 10^7^ cells were harvested, and the total RNA and lipids were extracted. The experiments were repeated either two or three times.

Human primary monocytes were isolated from healthy donors according to a previous study^20^. Briefly, blood was washed with phosphate-buffered saline (PBS) and spun at 250 *g* for 10 min to remove plasma and platelets. Then, the blood was loaded on a Ficoll gradient (Gelifesciense #17-1440-02) and spun at 400 *g* for 30 min according to the manufacturer’s instructions. The PBMC layer was collected, and the cells were washed two times in PBS and resuspended in RPMI 1640 supplemented with 20% FBS. The PBMCs were cultured for 2–4 h, and the cells remaining in suspension were then removed. The remaining adherent cells were considered monocytes. The monocytes were further cultured for 7 days under 20% FBS with the addition of 100 ng/ml M-CSF for macrophage differentiation. Then, the macrophages were polarized by removing the culture medium and culturing the cells for an additional 18 h in RPMI 1640 supplemented with 5% FBS and 100 ng/ml LPS (for M1 polarization) or 20 ng/ml IL-4 (for M2 polarization). All methods were carried out in accordance with the relevant guidelines and regulations. All experimental protocols were approved by the Department of Systems Biology for Medicine and the Institutes of Biomedical Sciences (Shanghai Medical College, Fudan University). Informed consent was obtained from all donors.

### RNA isolation and quantitative RT-PCR analysis

Total RNA was extracted using TRIzol (Invitrogen, USA) according to the manufacturer’s instructions. A PrimeScript RT Master Mix (TaKaRa, Japan) was used to generate cDNA, which was then quantified using SYBR Premix Ex Taq (TaKaRa, Japan). Real-time PCR was performed using a 7500 Real-Time PCR System (Applied Biosystems, USA). Individual gene expression was normalized to the housekeeping gene actin. Supplemental Table 2 lists the gene-specific primers. Macrophage marker primer sequences (Sangon Biotech Co., Ltd., China) were used in the qRT-PCR experiment.

### Sample preparation

A sample of 10^7^ cells was harvested, washed with PBS twice, and then freeze/thawed repeatedly in liquid nitrogen to lyse the cells. To extract total lipids from the cell lysate, 5 ml of methanol:chloroform:formic acid (v/v/v 10:10:1) solution was added to the lysate, mixed by vortexing, and then stored overnight at −20 °C. Subsequently, an internal standard (IS) cocktail (including six IS cocktails purchased from Avanti Lipids Polar, Inc., USA; PE, PC, PS, PG, PI, and d18Cer) and 2.2 ml of Hajra’s solution containing 0.2 mol/L phosphoricacid and 1 mol/L potassium chloride were added to the mixture, which was then centrifuged at 3,000 rpm for 5 min at room temperature. Two phases were obtained: an aqueous phase and an organic phase; the lipids were enriched in the organic phase. The extracts were dried under a weak nitrogen stream and stored at −20 °C. Quartz glass containers were used throughout the process to avoid polyethyleneglycol pollution.

### MRM-MS analysis

We adopted the method of interfacing HPLC with electrospray ionization-mass spectrometry (ESI-MS). Supplementary Fig. 5 lists the liquid chromatography separation conditions. Briefly, the lipid extracts were re-dissolved with the solution A phase (IPA:hexane:100 nmol/L AA) and loaded onto a silica column (2.1 × 250 mm, particle size: 5 µm, Welch Materials, Inc., USA) with a flow rate of 300 µL/min. The mobile phases A and B consisted of IPA:hexane:100 mM NH_4_COOH (v/v/v58:40:2) and IPA:hexane:100 mM NH_4_COOH (v/v/v 50:40:10), respectively. The B phase began at 50% and was held for 5 min. The linear gradient was increased to 100% B over the course of 30 min, held for 10 min and returned to the initial setup after 1 min. The MRM experiments were performed on a 4000 QTRAP hybrid triple quadrupole/linear ion trap mass spectrometer (AB SCIEX, CA, USA). Table 2 lists the parameters of the MRM method. Supplementary Table S1 lists the MRM transitions (precursor ion/product ion) and collision energy (CE) values selected for each individual lipid.

**Table 2:** Parameters of the MRM methods.

Parameter	Mode	
	Positive ion mode Negative ion mode	
Ion source	Turbo spray	Turbo spray
Curtain gas	20.0	20.0
Collision gas	low	low
Ion spray voltage (V)	5500.0	−4500.0
Temperature (°C)	450.0	450.0
Ion source gas	20.0	20.0
Declustering potential (DP)	60.0	−100.0
Entrance potential (EP)	10.0	−10.0
Collision cell exit potential (CXP)	15.0	−15.0

### Data analysis

Analyst^®^ software (Version 1.6, AB SCIEX) was used to collect and analyze the HPLC-MS/MS data. PCA was used to visualize the general clustering between the control and model groups. PLS-DA was used to identify the different lipids responsible for the separation. The level of significance of the difference between the data sets was determined using unpaired two-tailed Student’s t-tests (*p < 0.05,**p < 0.01,***p < 0.001). The results are expressed as the mean ± SEM. Three independent biological experiments were generally analyzed to produce the plots shown in Figs 2, 3, 4 and 5.

## Electronic supplementary material

Quantitative profiling of glycerophospholipids during mouse and human macrophage differentiation using targeted mass spectrometry
