# Supplementary material for: Quantitative profiling of glycerophospholipids during mouse and human macrophage differentiation using targeted mass spectrometry
Source: Sci Rep. 2017 Mar 24;7:412. doi: 10.1038/s41598-017-00341-2 (PMC5428309; doi:10.1038/s41598-017-00341-2)
Supplement: Supplementary file 1 — Quantitative profiling of glycerophospholipids during mouse and human macrophage differentiation using targeted mass spectrometry [file 41598_2017_341_MOESM1_ESM.pdf]

Supplementary Information

Quantitative profiling of glycerophospholipids during mouse and human macrophage differentiation using targeted mass spectrometry

Cuiping Zhang[1*], Yi Wang[2*], Fang Wang[2], Zhenxin Wang[2], Yu Lu[1], Ying Xu[2], Ke Wang[2], Huali Shen[2], Pengyuan Yang[2], Shan Li[1‡], Xue Qin[1‡], Hongxiu Yu[2‡]

[1]Department of Clinical Laboratory, First Affiliated Hospital of Guangxi Medical University, Nanning 530021,Guangxi, China.
[2] Department of Systems Biology for Medicine and Institutes of Biomedical Sciences, Shanghai Medical College,Fudan University, Shanghai 200032, China.
*These authors contributed equally to this work.
‡Correspondingauthors: Shan Li (lis8858@126.com), Xue Qin (qinxue919@126.com) Hongxiu Yu (hongxiuyu@fudan.edu.cn)

Table S1. Identification of lipid species by target-MS based on MRM method

Class	Subclass	Lipids	Transitions (*m/z*)	RT[#] (min)	CE[*] (ev)	Stability RSD%	Ion mode
GL	PC	PC30:0(14:0/16:0)	706.5 / 184.1	43.63	25	2.90	positive
		PC30:1(14:0/16:1)	704.5 / 184.1	46.2	25	3.30	positive
		PC31:0(16:0e/16:0)	720.5 / 184.1	41.81	25	2.39	positive
		PC31:1p(16:0p/16:0)	718.5 / 184.1	41.92	25	0.04	positive
		PC32:0(16:0/16:0)	734.6 / 184.1	42.77	25	1.44	positive
		PC32:1(14:0/18:1)or(16:0/16:1)	732.5 / 184.1	42.88	25	1.33	positive
		PC32:2(14:0/18:2)or(16:1/16:1)	730.5 / 184.1	43.1	25	1.10	positive
		PC 33:0e(18:0e/16:0)	748.6 / 184.1	41.06	25	1.06	positive
		PC33:1e(18:0e/16:1)or(18:1e/16:0)	746.6 / 184.1	41.17	25	1.07	positive
		PC 33:2e(16:1e/18:1)	744.6 / 184.1	41.38	25	1.31	positive
		PC 34:0(16:0/18:0)	762.6 / 184.1	42.13	25	0.57	positive
		PC 34:1(16:0/18:1)	760.6 / 184.1	42.24	25	0.58	positive
		PC34:2 (16:0/18:2)or(16:1/18:1)	758.6 / 184.1	42.56	25	0.12	positive
		PC34:3(16:0/18:3)or(16:1/18:2)	756.6 / 184.1	42.67	25	4.10	positive
		PC34:4(14:0/20:4)or(16:1/18:3)	754.5 / 184.1	42.88	25	2.06	positive
		PC 34:5(14:0/20:5)	752.5 / 184.1	43.31	25	11.10	positive
		PC35:1(16:0/19:1)or(17:0/18:1)or(17:1/18:0)	774.6 / 184.1	40.42	25	2.52	positive
		PC 35:2e(16:0e/20:2)	772.6 / 184.1	40.74	25	1.92	positive
		PC 35:3(17:1/18:2)	770.6 / 184.1	40.52	25	3.89	positive
		PC 35:4(15:0/20:4)	768.6 / 184.1	40.95	25	0.80	positive
		PC 35:5(15:0/20:5)	766.5 / 184.1	41.27	25	0.49	positive
		PC 36:0p(16:0p/20:0)	790.6 / 184.1	41.38	25	0.08	positive
		PC 36:1(18:0/18:1)	788.6 / 184.1	41.49	25	3.97	positive
		PC 36:2(18:0/18:2)or(18:1/18:1)	786.6 / 184.1	41.7	25	2.18	positive
		PC36:3(16:0/20:3)or(18:0/18:3)or(18:1/18:20)	784.6 / 184.1	42.02	25	2.98	positive
		PC36:4(16:0/20:4)	782.6 / 184.1	42.02	25	1.28	positive
		PC36:5 (16:1/20:4)	780.6 / 184.1	42.45	25	0.54	positive

GL	PC	PC 36:6(14:0/22:6)	778.5 / 184.1	42.77	25	7.48	positive
		PC 37:1(18:0/19:1)or(19:0/18:1)	802.6 / 184.1	39.67	25	0.47	positive
		PC 37:2(18:1/19:1)or(18:2/19:0)	800.6 / 184.1	44.17	25	3.90	positive
		PC 37:3(17:0/20:3)or(19:1/18:2)	798.6 / 184.1	40.1	25	0.26	positive
		PC 37:4(17:0/20:4)	796.6 / 184.1	40.1	25	1.52	positive
		PC 37:5e(16:0e/22:5)or(18:0e/20:5)	794.6 / 184.1	40.52	25	1.47	positive
		PC 37:6(15:0/22:6)	792.6 / 184.1	40.95	25	1.24	positive
		PC 38:1(18:1/20:0)	816.6 / 184.1	43.42	25	0.87	positive
		PC 38:2(16:0/22:2)	814.6 / 184.1	43.85	25	0.35	positive
		PC 38:3(18:0/20:3)or(18:1/20:2)	812.6 / 184.1	41.27	25	1.95	positive
		PC 38:4(18:0/20:4)	810.6 / 184.1	41.17	25	1.09	positive
		PC 38:5(18:0/20:5)	808.6 / 184.1	41.49	25	4.38	positive
		PC 38:6(18:2/20:4)	806.6 / 184.1	42.02	25	2.44	positive
		PC 38:7(16:1/22:6) or (18:2/20:5)	804.6 / 184.1	39.67	25	1.94	positive
		PC 39:3(19:0/20:3)	826.6 / 184.1	39.45	25	2.34	positive
		PC 39:6(17:0/22:6)	820.6 / 184.1	40.31	25	4.29	positive
		PC 39:7(17:1/22:6)	818.6 / 184.1	40.52	25	0.71	positive
		PC 40:1(18:1/22:0)	844.7 / 184.1	40.1	25	1.90	positive
		PC 40:4(18:0/22:4) or(20:1/20:3)	838.6 / 184.1	40.74	25	2.92	positive
		PC 40:5(18:0/22:5)	836.6 / 184.1	41.06	25	2.50	positive
		PC 40:6(18:0/22:6)	834.6 / 184.1	41.27	25	2.24	positive
		PC 40:7(18:1/22:6)	832.6 / 184.1	41.49	25	2.54	positive
		PC 40:8(20:4/20:4)	830.6 / 184.1	39.13	25	5.80	positive
		PC 41:6(19:0/22:6)	848.6 / 184.1	39.56	25	5.01	positive
	PE	PE 32:0(16:0/16:0)	692.5 / 551.5	19.52	25	1.43	positive
		PE 32:1(14:0/18:1)or(16:0/16:1)	690.5 / 549.5	19.52	25	0.28	positive
		PE 32:2(14:0/18:2)	688.5 / 547.5	19.62	25	0.00	positive
		PE 33:1(15:0/18:1)	704.6 / 563.6	17.48	25	8.28	positive
		PE 33:2(15:0/18:2)	702.5 / 561.5	17.44	25	0.11	positive
		PE 34:0(16:0/18:0)	720.6 / 579.6	18.98	25	0.95	positive
		PE 34:1(16:0/18:1)	718.5 / 577.5	18.98	25	0.22	positive
		PE 34:2(16:0/18:2)or(16:1/18:1)	716.5 / 575.5	18.98	25	0.56	positive
		PE 34:3(16:0/18:3)	714.5 / 573.5	19.3	25	1.83	positive

GL	PE	PE 35:1(17:0/18:1)	732.6 / 591.6	16.84	25	3.10	positive
		PE 35:2(17:0/18:2)	730.5 / 589.5	16.96	25	1.94	positive
		PE 35:3(17:1/18:2)	728.5 / 587.5	16.73	25	0.09	positive
		PE 35:4p(16:0p/20:3)or(16:0e/20:4)	726.5 / 585.5	17.05	25	0.07	positive
		PE 35:5p(16:0p/20:4)	724.5 / 583.5	17.37	25	0.84	positive
		PE 35:6p(16:0p/20:5)	722.5 / 581.5	18.87	25	1.83	positive
		PE 36:1(16:0/20:1)or(18:0/18:1)	746.6 / 605.6	18.34	25	3.49	positive
		PE 36:2(18:0/18:2)or(18:1/18:1)	744.6 / 603.6	18.45	25	4.51	positive
		PE 36:3(18:1/18:2)	742.5 / 601.5	18.77	25	7.72	positive
		PE 36:4(16:0/20:4)	740.5 / 599.5	18.55	25	3.94	positive
		PE 36:5(16:0/20:5)	738.5 / 597.5	18.77	25	5.43	positive
		PE 36:6(16:1/20:5)	736.5 / 595.5	19.2	25	4.03	positive
		PE 37:2(19:0/18:2)	758.6 / 617.6	16.2	25	5.77	positive
		PE 37:3(17:0/20:3)	756.6 / 615.6	16.52	25	3.06	positive
		PE 37:4(17:0/20:4)	754.5 / 613.5	16.41	25	2.90	positive
		PE 37:5p(16:0p/22:4) or(18:1p/20:3)	752.5 / 611.5	17.05	25	0.71	positive
		PE37:6p(18:0p/20:5)or(18:1p/20:4)or(16:0e/22:6)	750.5 / 609.5	16.95	25	1.09	positive
		PE 37:7p(16:0p/22:6)	748.6 / 607.6	18.34	25	2.06	positive
		PE 38:1(18:0/20:1)	774.6 / 633.6	17.59	25	5.78	positive
		PE 38:2(18:1/20:1)	772.6 / 631.6	17.59	25	3.66	positive
		PE 38:3(18:0/20:3)	770.6 / 629.6	17.48	25	1.96	positive
		PE 38:4(16:0/22:4)	768.6 / 627.6	17.8	25	3.33	positive
		PE 38:5(18:0/20:5)	766.5 / 625.5	18.02	25	3.05	positive
		PE 38:6(16:0/22:6)or(16:1/22:5)or(20:2/18:4)	764.5 / 623.5	18.66	25	3.34	positive
		PE 38:7(16:1/22:6)or(18:2/20:5)	762.5 / 621.5	18.77	25	6.75	positive
		PE 40:1(22:0/18:1)	802.6 / 661.6	17.05	25	1.69	positive
		PE 40:2(18:1/22:1)	800.6 / 659.6	17.16	25	1.77	positive
		PE 40:3(18:1/22:2) or (22:1/18:2)	822.6 / 681.6	17.16	25	3.81	positive
		PE 40:4(20:0/20:4)	818.6 / 677.6	17.37	25	0.05	positive
		PE 40:5(18:0/22:5)	816.6 / 675.6	17.80	25	2.50	positive
		PE 40:6(18:0/22:6)	792.6 / 651.6	17.91	25	4.50	positive
		PE 40:7(18:1/22:6)	790.5 / 649.5	18.12	25	2.54	positive
		PE 42:5; PE 22:1/20:4	822.6 / 681.6	17.16	25	8.28	positive

GL		PE 42:7; PE 20:1/22:6	818.6 / 677.6	17.37	25	8.11	positive
		PE 42:8; PE 20:2/22:6	816.6 / 675.6	17.8	25	0.31	positive
	PG	PG30:0(14:0/16:0)	695.5 / 523.5	12.23	25	3.30	positive
		PG30:1(14:0/16:1)	693.5 / 521.5	12.23	25	3.85	positive
		PG31:0(16:0e/16:0)	709.5 / 537.5	11.8	25	6.14	positive
		PG31:1(16:0p/16:0)	707.5 / 535.5	12.12	25	13.32	positive
		PG32:0(16:0/16:0)	723.5 / 551.5	12.02	25	2.18	positive
		PG32:1(14:0/18:1)or(16:0/16:1)	721.5 / 549.5	12.02	25	1.96	positive
		PG32:2(14:0/18:2)	719.5 / 547.5	12.12	25	42.71	positive
		PG33:0(18:0e/16:0)	737.5 / 565.5	12.02	25	2.52	positive
		PG33:1(15:0/18:1)	735.5 / 563.5	11.91	25	0.33	positive
		PG33:2(15:0/18:2)	733.5 / 561.5	12.02	25	0.43	positive
		PG34:0(16:0/18:0)	751.5 / 579.5	11.91	25	0.39	positive
		PG34:1(16:0/18:1)	749.5 / 577.5	11.91	25	0.74	positive
		PG34:2(16:0/18:2)or(16:1/18:1)	747.5 / 575.5	11.91	25	0.98	positive
		PG34:3(16:0/18:3)	745.5 / 573.5	12.02	25	4.63	positive
		PG34:4(14:0/20:4)or(16:1/18:3)	743.5 / 571.5	12.02	25	5.72	positive
		PG35:1(17:0/18:1)	763.5 / 591.5	11.8	25	0.30	positive
		PG35:2(17:0/18:2)	761.5 / 589.5	11.8	25	4.60	positive
		PG35:5(15:0/20:5)	755.5 / 583.5	11.48	25	16.86	positive
		PG36:0 p(16:0p/20:0)	779.5 / 607.5	11.69	25	10.43	positive
		PG36:1(16:0/20:1)or(18:0/18:1)	777.5 / 605.5	11.69	25	7.30	positive
		PG36:2(18:0/18:2)or(18:1/18:1)	775.5 / 603.5	11.8	25	6.13	positive
		PG36:3(18:1/18:2)	773.5 / 601.5	11.8	25	2.44	positive
		PG36:4(16:0/20:4)	771.5 / 599.5	11.8	25	0.07	positive
		PG36:5(16:0/20:5)	769.5 / 597.5	11.91	25	6.15	positive
		PG36:6(16:1/20:5)	767.5 / 595.5	6.12	25	10.88	positive
		PG37:3(17:0/20:3)	787.5 / 615.5	6.12	25	0.50	positive
		PG37:4(17:0/20:4)	785.5 / 613.5	11.48	25	0.69	positive
		PG37:5 e(16:0e/22:5)or(18:0e/20:5)	783.5 / 611.5	6.12	25	27.25	positive
		PG37:6p(18:0p/20:5)or(18:1p/20:4)or(16:0e/22:6)	781.5 / 609.5	11.8	25	10.44	positive
		PG38:1(18:0/20:1)	805.5 / 633.5	11.59	25	4.99	positive
		PG38:2(18:1/20:1)	803.5 / 631.5	11.59	25	0.02	positive

GL	PG	PG38:3(18:0/20:3)	801.5 / 629.5	11.59	25	1.74	positive
		PG38:4(16:0/22:4)	799.5 / 627.5	11.69	25	3.86	positive
		PG38:5(18:0/20:5)	797.5 / 625.5	11.69	25	9.29	positive
		PG38:6(16:0/22:6)or(16:1/22:5)or(20:2/18:4)	795.5 / 623.5	11.8	25	6.63	positive
		PG39:3(19:0/20:3)	815.5 / 643.5	6.55	25	2.96	positive
		PG39:6(17:0/22:6)	809.5 / 637.5	12.55	25	2.30	positive
		PG39:7(17:1/22:6)	807.5 / 635.5	11.59	25	12.80	positive
		PG40:1(18:1/22:0)	833.5 / 661.5	11.37	25	0.39	positive
		PG40:4(18:0/22:4) or (20:0/20:4)	827.5 / 655.5	11.59	25	0.29	positive
		PG40:5(18:0/22:5)	825.5 / 653.5	11.69	25	11.00	positive
		PG40:6(18:0/22:6)	823.5 / 651.5	11.59	25	3.67	positive
		PG40:7(18:1/22:6)	821.5 / 649.5	9.34	25	2.26	positive
		PG40:8(20:4/20:4)	819.5 / 647.5	11.69	25	3.22	positive
		PG41:6(19:0/22:6)	837.5 / 665.5	6.55	25	1.27	positive
	PS	PS30:0(14:0/16:0)	708.5 / 523.5	26.38	25	4.52	positive
		PS30:1(14:0/16:1)	706.5 / 521.5	26.59	25	0.37	positive
		PS31:0(16:0e/16:0)	722.5 / 537.5	26.06	25	2.24	positive
		PS31:1(16:0p/16:0)	720.5 / 535.5	24.34	25	8.40	positive
		PS32:0(16:0/16:0)	736.5 / 551.5	25.73	25	5.02	positive
		PS32:1(14:0/18:1)or(16:0/16:1)	734.5 / 549.5	25.73	25	2.21	positive
		PS32:2(14:0/18:2)	732.5 / 547.5	25.95	25	4.98	positive
		PS33:0(18:0e/16:0)	750.5 / 565.5	25.52	25	3.45	positive
		PS33:1(15:0/18:1)	748.5 / 563.5	23.91	25	0.21	positive
		PS33:2(15:0/18:2)	746.5 / 561.5	24.02	25	9.37	positive
		PS34:0(15:0/18:2)	764.5 / 579.5	25.2	25	6.77	positive
		PS34:1(16:0/18:1)	762.5 / 577.5	25.2	25	2.41	positive
		PS34:2(16:0/18:2)or(16:1/18:1)	760.5 / 575.5	25.31	25	3.74	positive
		PS34:3(16:0/18:3)	758.5 / 573.5	25.52	25	3.39	positive
		PS34:4(14:0/20:4)or(16:1/18:3)	756.5 / 571.5	25.41	25	5.44	positive
		PS35:1(17:0/18:1)	776.5 / 591.5	24.88	25	3.22	positive
		PS35:2(17:0/18:2)	774.5 / 589.5	23.53	25	4.77	positive
		PS35:3(17:1/18:2)	772.5 / 587.5	23.91	25	4.57	positive
		PS35:4(15:0/20:4)	770.5 / 585.5	23.7	25	6.22	positive

**GL**	**PS**	PS35:5(15:0/20:5)	768.5 / 583.5	31.74	25	14.86	positive
		PS36:0 p(16:0p/20:0)	792.5 / 607.5	24.45	25	2.06	positive
		PS36:1(16:0/20:1)or(18:0/18:1)	790.5 / 605.5	24.45	25	1.18	positive
		PS36:2(18:0/18:2)or(18:1/18:1)	788.5 / 603.5	24.88	25	0.65	positive
		PS36:3(18:1/18:2)	786.5 / 601.5	24.88	25	2.16	positive
		PS36:4(16:0/20:4)	784.5 / 599.5	24.88	25	4.59	positive
		PS36:5(16:0/20:5)	782.5 / 597.5	25.2	25	3.13	positive
		PS36:6(16:1/20:5)	780.5 / 595.5	24.98	25	2.94	positive
		PS37:1(18:0/19:1)or(19:0/18:1)	804.5 / 619.5	24.23	25	2.12	positive
		PS37:2(18:1/19:1)or(18:2/19:0)	802.5 / 617.5	24.34	25	3.17	positive
		PS37:3(17:0/20:3)or(19:1/18:2)	800.5 / 615.5	23.14	25	4.75	positive
		PS37:4(17:0/20:4)	798.5 / 613.5	23.38	25	0.48	positive
		PS37:5e(16:0e/22:5)or(18:0e/20:5)	796.5 / 611.5	23.59	25	11.98	positive
		PS37:6(15:0/22:6)	794.5 / 609.5	24.55	25	8.69	positive
		PS38:1(18:1/20:0)	818.5 / 633.5	24.02	25	7.62	positive
		PS38:2(16:0/22:2)	816.5 / 631.5	24.02	25	6.94	positive
		PS38:3(18:0/20:3)	814.5 / 629.5	24.23	25	0.05	positive
		PS38:4(18:0/20:4)	812.5 / 627.5	24.13	25	1.64	positive
		PS38:5(18:0/20:5)	810.5 / 625.5	24.34	25	1.52	positive
		PS38:6 (18:2/20:4)	808.5 / 623.5	24.88	25	0.62	positive
		PS38:7(16:1/22:6) or (18:2/20:5)	806.5 / 621.5	24.23	25	6.08	positive
		PS39:3(19:0/20:3)	828.5 / 643.5	23.91	25	8.80	positive
		PS39:6(17:0/22:6)	822.5 / 637.5	24.45	25	2.57	positive
		PS39:7(17:1/22:6)	820.5 / 635.5	24.02	25	2.58	positive
		PS40:1(18:1/22:0)	776.5 / 591.5	24.88	25	3.75	positive
		PS40:4(18:0/22:4) or(20:1/20:3)	774.5 / 589.5	23.53	25	0.76	positive
		PS40:5(18:0/22:5)	772.5 / 587.5	23.91	25	1.59	positive
		PS40:6(18:0/22:6)	770.5 / 585.5	23.7	25	1.73	positive
		PS40:7(18:1/22:6)	768.5 / 583.5	31.74	25	1.85	positive
		PS40:8(20:4/20:4)	792.5 / 607.5	24.45	25	8.95	positive
		PS41:6(19:0/22:6)	790.5 / 605.5	24.45	25	5.60	positive
		lysoPC 14:0 (sn-2)	468.3 / 184.1	54.5	25	0.86	positive
		lysoPC 15:0 (sn-2)	482.3 / 184.1	52.96	25	2.84	positive

**GL**	**lysoPC**	lysoPC 15:1 (sn-1)	480.3 / 184.1	53.6	25	7.68	positive
		lysoPC 16:0 (sn-2)	496.3 / 184.1	51.88	25	4.54	positive
		lysoPC 16:1 (sn-1)	494.3 / 184.1	52.85	25	5.31	positive
		lysoPC 17:0 (sn-1)	510.4 / 184.1	51.35	25	6.32	positive
		lysoPC 17:1 (sn-1)	508.3 / 184.1	51.99	25	4.52	positive
		lysoPC 18:0 (sn-1)	524.4 / 184.1	51.03	25	9.37	positive
		lysoPC 18:1 (sn-1)	522.4 / 184.1	51.03	25	5.94	positive
		lysoPC 18:2 (sn-1)	520.3 / 184.1	52.1	25	7.20	positive
		lysoPC 18:3 (sn-1)	518.3 / 184.1	52.85	25	7.56	positive
		lysoPC 19:0 (sn-1)	538.4 / 184.1	50.17	25	6.52	positive
		lysoPC 20:0 (sn-1)	552.4 / 184.1	49.85	25	0.88	positive
		lysoPC 20:1 (sn-1)	550.4 / 184.1	49.85	25	4.73	positive
		lysoPC 20:2 (sn-1)	548.4 / 184.1	50.38	25	1.86	positive
		lysoPC 20:3 (sn-1)	546.4 / 184.1	50.49	25	0.56	positive
		lysoPC 20:4 (sn-1)	544.3 / 184.1	51.46	25	0.95	positive
		lysoPC 20:5 (sn-1)	542.3 / 184.1	52.42	25	1.54	positive
		lysoPC 22:0 (sn-1)	580.4 / 184.1	51.03	25	1.31	positive
		lysoPC 22:4 (sn-1)	572.4 / 184.1	50.28	25	6.09	positive
		lysoPC 22:6 (sn-1)	568.3 / 184.1	51.35	25	1.49	positive
	**lysoPE**	lysoPE 14:0 (sn-2)	426.3 / 285.3	33.24	25	15.04	positive
		lysoPE 16:0 (sn-1)	454.3 / 313.3	31.82	25	1.27	positive
		lysoPE 17:0 (sn-2)	468.3 / 327.3	31.5	25	8.00	positive
		lysoPE 18:0 (sn-1)	482.3 / 341.3	32.38	25	14.75	positive
		lysoPE 18:1 (sn-1)	480.3 / 339.3	31.2	25	2.06	positive
		lysoPE 18:2 (sn-2)	478.3 / 337.3	31.74	25	5.19	positive
		lysoPE 20:0 (sn-2)	510.4 / 369.4	29.7	25	4.89	positive
		lysoPE 20:1 (sn-2)	508.3 / 367.3	29.91	25	4.51	positive
		lysoPE 20:3 (sn-2)	504.3 / 363.3	30.34	25	6.90	positive
		lysoPE 20:4 (sn-2)	502.3 / 361.3	30.88	25	8.26	positive
		lysoPE 20:5 (sn-1)	500.3 / 359.3	31.63	25	10.15	positive
		lysoPE 22:6 (sn-1)	526.3 / 385.3	31.09	25	7.80	positive
		Cer120	482.6 / 264.4	6.23	35	2.65	positive
		Cer160	538.7 / 264.4	6.23	37.5	8.37	positive

**Ceramide**	**Cer**	Cer180	566.7 / 264.4	6.23	37.5	9.86	positive
	**Cer**	Cer241	648.9 / 264.4	6.23	42.5	12.39	positive
		Cer240	650.9 / 264.4	6.23	42.5	6.02	positive
		Cer250	664.9 / 264.4	6.12	45	7.04	positive
**Ceramide**	**C1P**	C1P120	562.6 / 264.4	6.23	45	11.60	positive
		C1P160	618.7 / 264.4	6.23	47.5	2.39	positive
		C1P240	730.9 / 264.4	7.62	52.5	8.19	positive
**Sphingoid bases**	**So**	d171So	286.4 / 268.3	13.52	21	15.51	positive
		d181So	300.4 / 282.4	13.19	21	4.63	positive
	**Sa**	d170Sa	288.4 / 60.0	9.01	50	27.67	positive
		d180Sa	302.4 / 60.0	14.48	50	6.44	positive
	**S1P**	d171S1P	366.4 / 250.3	9.55	25	12.80	positive
		d18:1S1P	380.4 / 264.4	49.31	25	14.05	positive
	**Sa1P**	d170Sa1P	368.4 / 252.2	6.23	25	50.48	positive
		d18:0Sa1P	382.4 / 266.4	57.89	25	11.91	positive
**Phosphosphingolipids**	**SM**	SM120	647.7 / 184.4	47.38	40	1.70	positive
		SM180	731.8 / 184.4	42.88	45	1.40	positive
		SM240	815.9 / 184.4	43.31	50	1.93	positive
**GL**	**PI**	PI 30:0(14:0/16:0)	781.5 / 241.1	20.42	-65	2.73	negative
		PI 30:1(14:0/16:1)	779.5 / 241.1	20.65	-65	4.30	negative
		PI 31:0(16:0e/16:0)	795.5 / 241.1	21.09	-65	5.34	negative
		PI 31:1p(16:0p/16:0)	793.5 / 241.1	19.7	-65	5.97	negative
		PI 32:0(16:0/16:0)	809.5 / 241.1	20.58	-65	8.88	negative
		PI 32:1(14:0/18:1) or (16:0/16:1)	807.5 / 241.1	20.55	-65	3.92	negative
		PI 32:2(14:0/18:2)or (16:1/16:1)	805.5 / 241.1	20.71	-65	12.41	negative
		PI 33:0e(18:0e/16:0)	823.5 / 241.1	20.12	-65	0.49	negative
		PI 33:1e(18:0e/16:1) or (18:1e/16:0)	821.5 / 241.1	20.22	-65	0.50	negative
		PI 33:2e(16:1e/18:1)	819.5 / 241.1	20.28	-65	12.42	negative
		PI 34:0(16:0/18:0)	837.5 / 241.1	19.94	-65	0.67	negative
		PI 34:1(16:0/18:1)	835.5 / 241.1	19.95	-65	2.04	negative
		PI 34:2(16:0/18:2) or (16:1/18:1)	833.5 / 241.1	20.12	-65	2.86	negative
		PI 34:3(16:0/18:3) or (16:1/18:2)	831.5 / 241.1	20.12	-65	1.01	negative

**GL**	**PI**	PI 34:4(14:0/20:4)	829.5 / 241.1	20.48	-65	3.41	negative
		PI 34:5(14:0/20:5)	827.5 / 241.1	19.47	-65	0.76	negative
		PI35:1(16:0/19:1) or(17:0/18:1)or(17:1/18:0)	849.5 / 241.1	19.52	-65	6.85	negative
		PI 35:2e(16:0e/20:2)	847.5 / 241.1	19.82	-65	2.47	negative
		PI 35:3(17:1/18:2)	845.5 / 241.1	18.39	-65	5.20	negative
		PI 35:4(15:0/20:4)	843.5 / 241.1	18.69	-65	3.24	negative
		PI 35:5(15:0/20:5)	841.5 / 241.1	19.28	-65	8.17	negative
		PI 36:0p(16:0p/22:6)	865.5 / 241.1	19.32	-65	10.61	negative
		PI 36:1(18:0/18:1)	863.5 / 241.1	19.39	-65	10.18	negative
		PI 36:2(18:0/18:2) or (18:1/18:1)	861.5 / 241.1	19.5	-65	8.46	negative
		PI 36:4(16:0/20:4)	857.5 / 241.1	19.65	-65	2.21	negative
		PI36:5(14:0/22:5)or(16:0/20:5)or (16:1/20:4)	855.5 / 241.1	19.92	-65	9.86	negative
		PI 36:6(14:0/22:6)	853.5 / 241.1	19.75	-65	5.68	negative
		PI 37:1(18:0/19:1) or (19:0/18:1)	877.5 / 241.1	19.06	-65	7.16	negative
		PI 37:2(18:1/19:1) or (18:2/19:0)	875.5 / 241.1	19.00	-65	6.37	negative
		PI 37:3(17:0/20:3) or (19:1/18:2)	873.5 / 241.1	19.03	-65	0.54	negative
		PI 37:4(17:0/20:4)	871.5 / 241.1	18.08	-65	2.96	negative
		PI 37:5e(16:0e/22:5) or (18:0e/20:5)	869.5 / 241.1	18.33	-65	0.85	negative
		PI 37:6(15:0/22:6)	867.5 / 241.1	18.65	-65	12.62	negative
		PI 38:1(18:1/20:0)	891.5 / 241.1	18.67	-65	13.66	negative
		PI 38:2(16:0/22:2)	889.5 / 241.1	18.66	-65	5.99	negative
		PI 38:3(18:0/20:3) or (18:1/20:2)	887.5 / 241.1	18.66	-65	3.97	negative
		PI 38:4(18:0/20:4)	885.5 / 241.1	18.94	-65	4.14	negative
		PI 38:5(18:0/20:5)	883.5 / 241.1	19.21	-65	3.66	negative
		PI 38:6(16:0/22:6)or (18:2/20:4)	881.5 / 241.1	19.57	-65	4.38	negative
		PI 38:7(16:1/22:6) or (18:2/20:5)	879.5 / 241.1	20.03	-65	13.41	negative
		PI 39:3(19:0/20:3)	901.5 / 241.1	18.63	-65	13.42	negative
		PI 39:6(17:0/22:6)	895.5 / 241.1	18.24	-65	9.86	negative
		PI 39:7(17:1/22:6)	893.5 / 241.1	18.39	-65	5.62	negative
		PI 40:1(18:1/22:0)	919.5 / 241.1	18.18	-65	12.63	negative
		PI 40:4(18:0/22:4)or (20:1/20:3)	913.5 / 241.1	18.56	-65	1.19	negative
		PI 40:5(18:0/22:5)	911.5 / 241.1	18.85	-65	1.04	negative
		PI 40:6(18:0/22:6)	909.5 / 241.1	19.06	-65	1.08	negative

<table>
<tr><td rowspan="35">GL</td><td rowspan="3"></td><td>PI 40:7(18:1/22:6)</td><td>907.5 / 241.1</td><td>19.18</td><td>-65</td><td>2.08</td><td>negative</td></tr>
<tr><td>PI 40:8(20:4/20:4)</td><td>905.5 / 241.1</td><td>19.15</td><td>-65</td><td>9.33</td><td>negative</td></tr>
<tr><td>PI 41:6(19:0/22:6)</td><td>923.5 / 241.1</td><td>18.57</td><td>-65</td><td>5.70</td><td>negative</td></tr>
<tr><td rowspan="32">PG</td><td>PG30:0(14:0/16:0)</td><td>693.5 / 227.0</td><td>12.27</td><td>-35</td><td>5.87</td><td>negative</td></tr>
<tr><td>PG30:1(14:0/16:1)</td><td>691.5 / 227.0</td><td>12.27</td><td>-35</td><td>4.52</td><td>negative</td></tr>
<tr><td>PG31:0(16:0e/16:0)</td><td>707.5 / 227.0</td><td>9.91</td><td>-35</td><td>1.09</td><td>negative</td></tr>
<tr><td>PG31:1 p(16:0p/16:0)</td><td>705.5 / 227.0</td><td>12.15</td><td>-35</td><td>2.97</td><td>negative</td></tr>
<tr><td>PG32:0(16:0/16:0)</td><td>721.5 / 227.0</td><td>9.87</td><td>-35</td><td>0.27</td><td>negative</td></tr>
<tr><td>PG32:1(14:0/18:1) or (16:0/16:1)</td><td>719.5 / 227.0</td><td>10</td><td>-35</td><td>0.52</td><td>negative</td></tr>
<tr><td>PG32:2(14:0/18:2)or (16:1/16:1)</td><td>717.5 / 227.0</td><td>9.9</td><td>-35</td><td>13.64</td><td>negative</td></tr>
<tr><td>PG33:0 e(18:0e/16:0)</td><td>735.5 / 227.0</td><td>10.02</td><td>-35</td><td>17.29</td><td>negative</td></tr>
<tr><td>PG33:1 e(18:0e/16:1) or (18:1e/16:0)</td><td>733.5 / 227.0</td><td>7.27</td><td>-35</td><td>16.92</td><td>negative</td></tr>
<tr><td>PG33:2 e(16:1e/18:1)</td><td>731.5 / 227.0</td><td>9.39</td><td>-35</td><td>7.84</td><td>negative</td></tr>
<tr><td>PG34:0(16:0/18:0)</td><td>749.5 / 227.0</td><td>9.94</td><td>-35</td><td>4.00</td><td>negative</td></tr>
<tr><td>PG34:1(16:0/18:1)</td><td>747.5 / 227.0</td><td>10</td><td>-35</td><td>0.92</td><td>negative</td></tr>
<tr><td>PG34:2(16:0/18:2) or (16:1/18:1)</td><td>745.5 / 227.0</td><td>10.01</td><td>-35</td><td>1.11</td><td>negative</td></tr>
<tr><td>PG34:3(16:0/18:3) or (16:1/18:2)</td><td>743.5 / 227.0</td><td>9.98</td><td>-35</td><td>1.13</td><td>negative</td></tr>
<tr><td>PG34:4(14:0/20:4)</td><td>741.5 / 227.0</td><td>9.9</td><td>-35</td><td>2.66</td><td>negative</td></tr>
<tr><td>PG34:5(14:0/20:5)</td><td>739.5 / 227.0</td><td>9.9</td><td>-35</td><td>5.42</td><td>negative</td></tr>
<tr><td>PG35:1(16:0/19:1)or(17:0/18:1)</td><td>761.5 / 227.0</td><td>9.99</td><td>-35</td><td>2.14</td><td>negative</td></tr>
<tr><td>PG35:2 e(16:0e/20:2)</td><td>759.5 / 227.0</td><td>10.02</td><td>-35</td><td>3.73</td><td>negative</td></tr>
<tr><td>PG35:3(17:1/18:2)</td><td>757.5 / 227.0</td><td>9.97</td><td>-35</td><td>15.30</td><td>negative</td></tr>
<tr><td>PG35:4(15:0/20:4)</td><td>755.5 / 227.0</td><td>9.98</td><td>-35</td><td>7.84</td><td>negative</td></tr>
<tr><td>PG35:5(15:0/20:5)</td><td>753.5 / 227.0</td><td>10.02</td><td>-35</td><td>9.38</td><td>negative</td></tr>
<tr><td>PG36:0 p(16:0p/22:6)</td><td>777.5 / 227.0</td><td>10</td><td>-35</td><td>5.86</td><td>negative</td></tr>
<tr><td>PG36:1(18:0/18:1)</td><td>775.5 / 227.0</td><td>9.93</td><td>-35</td><td>10.60</td><td>negative</td></tr>
<tr><td>PG36:2(18:0/18:2) or (18:1/18:1)</td><td>773.5 / 227.0</td><td>9.88</td><td>-35</td><td>8.02</td><td>negative</td></tr>
<tr><td>PG36:3(18:1/18:2)</td><td>771.5 / 227.0</td><td>9.97</td><td>-35</td><td>0.67</td><td>negative</td></tr>
<tr><td>PG36:4(16:0/20:4) or (16:1/20:3)</td><td>769.5 / 227.0</td><td>10.02</td><td>-35</td><td>0.81</td><td>negative</td></tr>
<tr><td>PG36:5(14:0/22:5)or(16:0/20:5)or16:1/20:4</td><td>767.5 / 227.0</td><td>10.53</td><td>-35</td><td>1.28</td><td>negative</td></tr>
<tr><td>PG36:6(14:0/22:6)</td><td>765.5 / 227.0</td><td>9.95</td><td>-35</td><td>1.17</td><td>negative</td></tr>
<tr><td>PG37:1(18:0/19:1) or (19:0/18:1)</td><td>789.5 / 227.0</td><td>9.9</td><td>-35</td><td>7.14</td><td>negative</td></tr>
<tr><td>PG37:2(18:1/19:1) or (18:2/19:0)</td><td>787.5 / 227.0</td><td>9.95</td><td>-35</td><td>1.33</td><td>negative</td></tr>
</table>

**GL**	**PG**	PG37:3(17:0/20:3) or (19:1/18:2)	785.5 / 227.0	9.93	-35	13.97	negative
		PG37:4(17:0/20:4)	783.5 / 227.0	10.02	-35	5.04	negative
		PG37:5 e(16:0e/22:5) or (18:0e/20:5)	781.5 / 227.0	9.9	-35	0.20	negative
		PG37:6(15:0/22:6)	779.5 / 227.0	9.88	-35	0.75	negative
		PG38:1(18:1/20:0)	803.5 / 227.0	9.92	-35	3.19	negative
		PG38:2(16:0/22:2)	801.5 / 227.0	10.02	-35	5.64	negative
		PG38:3(18:0/20:3) or (18:1/20:2)	799.5 / 227.0	9.95	-35	3.75	negative
		PG38:4(18:0/20:4)	797.5 / 227.0	9.92	-35	3.49	negative
		PG38:5(18:0/20:5)	795.5 / 227.0	9.96	-35	3.28	negative
		PG38:6 (18:2/20:4)	793.5 / 227.0	9.98	-35	13.02	negative
		PG38:7(16:1/22:6) or (18:2/20:5)	791.5 / 227.0	6.1	-35	1.06	negative
		PG39:3(19:0/20:3)	813.5 / 227.0	10.06	-35	1.43	negative
		PG39:6(17:0/22:6)	807.5 / 227.0	9.93	-35	0.81	negative
		PG39:7(17:1/22:6)	805.5 / 227.0	10.4	-35	7.70	negative
		PG40:1(18:1/22:0)	831.5 / 227.0	10.02	-35	25.30	negative
		PG40:4(18:0/22:4)or (20:1/20:3)	825.5 / 227.0	9.96	-35	24.22	negative
		PG40:5(18:0/22:5)	823.5 / 227.0	10.02	-35	14.86	negative
		PG40:6(18:0/22:6)	821.5 / 227.0	9.9	-35	3.19	negative
		PG40:7(18:1/22:6)	819.5 / 227.0	5.98	-35	3.07	negative
		PG40:8(20:4/20:4)	817.5 / 227.0	10.02	-35	3.76	negative
		PG41:6(19:0/22:6)	835.5 / 227.0	9.98	-35	8.98	negative
	**PS**	PS30:0(14:0/16:0)	706.5 / 619.5	26.49	-25	12.94	negative
		PS30:1(14:0/16:1)	704.5 / 617.5	26.63	-25	6.92	negative
		PS31:0(16:0e/16:0)	720.5 / 633.5	26.2	-25	8.17	negative
		PS31:1(16:0p/16:0)	718.5 / 631.5	24.39	-25	4.72	negative
		PS32:0(16:0/16:0)	734.5 / 647.5	25.87	-25	2.24	negative
		PS32:1(14:0/18:1)or(16:0/16:1)	732.5 / 645.5	25.86	-25	2.36	negative
		PS32:2(14:0/18:2)	730.5 / 643.5	25.98	-25	0.07	negative
		PS33:0(18:0e/16:0)	748.5 / 661.5	25.57	-25	10.64	negative
		PS33:1(15:0/18:1)	746.5 / 659.5	25.56	-25	11.19	negative
		PS33:2(15:0/18:2)	744.5 / 657.5	24.03	-25	4.18	negative
		PS34:0(15:0/18:2)	762.5 / 675.5	25.23	-25	3.91	negative
		PS34:1(16:0/18:1)	760.5 / 673.5	25.23	-25	5.26	negative

GL	PS	PS34:2(16:0/18:2)or(16:1/18:1)	758.5 / 671.5	25.39	-25	0.16	negative
		PS34:3(16:0/18:3)	756.5 / 669.5	25.58	-25	2.81	negative
		PS34:4(14:0/20:4)or(16:1/18:3)	754.5 / 667.5	25.78	-25	12.60	negative
		PG34:5(14:0/20:5)	752.5 / 665.5	28.41	-25	ND	negative
		PS35:1(17:0/18:1)	774.5 / 687.5	24.96	-25	0.73	negative
		PS35:2(17:0/18:2)	772.5 / 685.5	25.08	-25	2.52	negative
		PS35:3(17:1/18:2)	770.5 / 683.5	25.28	-25	4.32	negative
		PS35:4(15:0/20:4)	768.5 / 681.5	23.84	-25	3.94	negative
		PS35:5(15:0/20:5)	766.5 / 679.5	23.91	-25	6.69	negative
		PS36:0 p(16:0p/20:0)	790.5 / 703.5	24.56	-25	0.25	negative
		PS36:1(16:0/20:1)or(18:0/18:1)	788.5 / 701.5	24.58	-25	0.40	negative
		PS36:2(18:0/18:2)or(18:1/18:1)	786.5 / 699.5	24.78	-25	2.35	negative
		PS36:3(18:1/18:2)	784.5 / 697.5	25	-25	2.91	negative
		PS36:4(16:0/20:4)	782.5 / 695.5	24.89	-25	3.96	negative
		PS36:5(16:1/20:4)	780.5 / 693.5	25.45	-25	4.76	negative
		PS36:6(16:1/20:5)	778.5 / 691.5	24.72	-25	0.30	negative
		PS37:1(18:0/19:1)or(19:0/18:1)	802.5 / 715.5	24.3	-25	2.94	negative
		PS37:2(18:1/19:1)or(18:2/19:0)	800.5 / 713.5	24.51	-25	6.05	negative
		PS37:3(17:0/20:3)or(19:1/18:2)	798.5 / 711.5	24.79	-25	4.57	negative
		PS37:4(17:0/20:4)	796.5 / 709.5	24.55	-25	2.97	negative
		PS37:5e(16:0e/22:5)or(18:0e/20:5)	794.5 / 707.5	23.68	-25	6.62	negative
		PS37:6(15:0/22:6)	792.5 / 705.5	24.64	-25	1.17	negative
		PS38:1(18:1/20:0)	816.5 / 729.5	24.06	-25	4.94	negative
		PS38:2(16:0/22:2)	814.5 / 727.5	24.16	-25	3.53	negative
		PS38:3(18:0/20:3)	812.5 / 725.5	24.32	-25	5.10	negative
		PS38:4(18:0/20:4)	810.5 / 723.5	24.22	-25	1.39	negative
		PS38:5(18:0/20:5)	808.5 / 721.5	24.68	-25	10.06	negative
		PS38:6(16:0/22:6) or (18:2/20:4)	806.5 / 719.5	24.94	-25	11.36	negative
		PS38:7(16:1/22:6) or (18:2/20:5)	804.5 / 717.5	24.46	-25	8.55	negative
		PS39:3(19:0/20:3)	826.5 / 739.5	23.95	-25	1.72	negative
		PS39:6(17:0/22:6)	820.5 / 733.5	24.69	-25	1.66	negative
		PS39:7(17:1/22:6)	818.5 / 731.5	24.05	-25	6.62	negative
		PS40:1(18:1/22:0)	844.5 / 757.5	23.52	-25	6.89	negative

GL		PS40:4(18:0/22:4) or(20:1/20:3)	838.5 / 751.5	23.98	-25	7.35	negative
		PS40:5(18:0/22:5)	836.5 / 749.5	24.16	-25	0.16	negative
		PS40:6(18:0/22:6)	834.5 / 747.5	24.23	-25	1.47	negative
		PS40:7(18:1/22:6)	832.5 / 745.5	24.44	-25	8.20	negative
		PS40:8(20:4/20:4)	830.5 / 743.5	23.93	-25	10.51	negative
		PS41:6(19:0/22:6)	848.5 / 761.5	24.07	-25	14.33	negative
	PA	PA 30:0(18:0/12:0)	619.4 / 199.2	27.03	-50	10.30	negative
		PA 30:0(16:0/14:0)	619.4 / 227.2	29.77	-50	1.60	negative
		PA 30:1(16:1/14:0)	617.4 / 153.0	29.16	-50	2.56	negative
		PA 31:0(16:0e/16:0)	633.4 / 153.0	23.28	-50	14.13	negative
		PA 31:1p(16:0p/16:0)	631.4 / 153.0	20.28	-50	5.59	negative
		PA 32:0(16:0/16:0)	647.4 / 153.0	28.54	-50	4.31	negative
		PA 32:1(14:0/18:1) or (16:0/16:1)	645.4 / 153.0	22.6	-50	3.20	negative
		PA 32:2(14:0/18:2) or (16:1/16:1)	643.4 / 153.0	28.25	-50	13.11	negative
		PA 33:0e(18:0e/16:0)	661.4 / 153.0	37.04	-50	1.68	negative
		PA 33:1e(18:0e/16:1) or (18:1e/16:0)	659.4 / 153.0	28.79	-50	1.55	negative
		PA 33:2e(16:1e/18:1)	657.4 / 153.0	40.17	-50	11.75	negative
		PA 34:0(16:0/18:0)	675.4 / 153.0	22.04	-50	2.82	negative
		PA 34:1(16:0/18:1)	673.4 / 153.0	22.08	-50	6.39	negative
		PA 34:2(16:0/18:2) or (16:1/18:1)	671.4 / 153.0	22.3	-50	12.84	negative
		PA 34:3(16:0/18:3) or (16:1/18:2)	669.4 / 153.0	22.53	-50	19.38	negative
		PA 34:4(14:0/20:4) or (16:1/18:3)	667.4 / 153.0	37.42	-50	25.92	negative
		PA 34:5(14:0/20:5)	665.4 / 153.0	37.79	-50	ND	negative
		PA35:1(16:0/19:1)or(17:0/18:1)or(17:1/18:0)	687.4 / 153.0	21.74	-50	8.93	negative
		PA 35:2e(16:0e/20:2)	685.4 / 153.0	22.03	-50	8.88	negative
		PA 35:3(17:1/18:2)	683.4 / 153.0	45.18	-50	0.89	negative
		PA 35:4(15:0/20:4)	681.4 / 153.0	0.26	-50	7.83	negative
		PA 35:5(15:0/20:5)	679.4 / 153.0	25.28	-50	7.57	negative
		PA 36:0p(16:0p/20:0)	703.4 / 153.0	21.53	-50	2.90	negative
		PA 36:1(18:0/18:1)	701.4 / 153.0	21.49	-50	2.75	negative
		PA 36:2(18:0/18:2) or (18:1/18:1)	699.4 / 153.0	21.64	-50	5.63	negative
		PA36:3(16:0/20:3)or(18:0/18:3)or(18:1/18:2)	697.4 / 153.0	21.66	-50	3.79	negative
		PA 36:4(16:0/20:4) or (16:1/20:3)	695.4 / 153.0	21.87	-50	7.52	negative

GL	PA	PA36:5(14:0/22:5)or(16:0/20:5)or(16:1/20:4)	693.4 / 153.0	25.28	-50	12.47	negative
		PA 36:6(14:0/22:6)	691.4 / 153.0	21.41	-50	8.33	negative
		PA 37:1; PA 18:0/19:1 or 19:0/18:1	715.4 / 153.0	21.5	-50	12.24	negative
		PA 37:2(18:1/19:1) or (18:2/19:0)	713.4 / 153.0	21.51	-50	7.95	negative
		PA 37:3(17:0/20:3) or (19:1/18:2)	711.4 / 153.0	21.22	-50	4.77	negative
		PA 37:4(17:0/20:4)	709.4 / 153.0	9.52	-50	10.53	negative
		PA 37:5e(16:0e/22:5) or (18:0e/20:5)	707.4 / 153.0	10.9	-50	24.42	negative
		PA 37:6(15:0/22:6)	705.4 / 153.0	21.15	-50	11.94	negative
		PA 38:1(18:1/20:0)	729.4 / 153.0	20.87	-50	1.00	negative
		PA 38:2(16:0/22:2)	727.4 / 153.0	21.17	-50	6.61	negative
		PA 38:3(18:0/20:3) or (18:1/20:2)	725.4 / 153.0	21.3	-50	6.37	negative
		PA 38:4(18:0/20:4)	723.4 / 153.0	21.22	-50	3.28	negative
		PA 38:5(18:0/20:5)	721.4 / 153.0	21.61	-50	4.04	negative
		PA 38:6(16:0/22:6) or (18:2/20:4)	719.4 / 153.0	22.03	-50	9.46	negative
		PA 38:7(16:1/22:6) or (18:2/20:5)	717.4 / 153.0	23.28	-50	7.11	negative
		PA 39:3(19:0/20:3)	739.4 / 153.0	20.87	-50	1.78	negative
		PA 39:6(17:0/22:6)	733.4 / 153.0	22.72	-50	12.08	negative
		PA 39:7(17:1/22:6)	731.4 / 153.0	21.03	-50	1.81	negative
		PA 40:1(18:1/22:0)	757.4 / 153.0	20.26	-50	2.61	negative
		PA 40:4(18:0/22:4) or (20:1/20:3)	751.4 / 153.0	20.87	-50	2.66	negative
		PA 40:5(18:0/22:5)	749.4 / 153.0	21.18	-50	7.11	negative
		PA 40:6(18:0/22:6)	747.4 / 153.0	21.2	-50	2.07	negative
		PA 40:7(18:1/22:6)	745.4 / 153.0	21.63	-50	3.52	negative
		PA 40:8(20:4/20:4)	743.4 / 153.0	25.04	-50	1.51	negative
		PA 41:6(19:0/22:6)	761.4 / 153.0	22.03	-50	10.01	negative
	lysoPI	lysoPI 14:0 (sn-2)	543.3 / 241.1	37.11	-65	3.75	negative
		lysoPI 15:0 (sn-2)	557.3 / 241.1	33.85	-65	11.68	negative
		lysoPI 15:1 (sn-1)	555.3 / 241.1	28.29	-65	3.76	negative
		lysoPI 16:0 (sn-2)	571.3 / 241.1	30.04	-65	9.17	negative
		lysoPI 16:1 (sn-1)	569.3 / 241.1	30.54	-65	8.88	negative
		lysoPI 17:0 (sn-1)	585.3 / 241.1	24.66	-65	8.57	negative
		lysoPI 17:1 (sn-1)	583.3 / 241.1	28.66	-65	7.28	negative
		lysoPI 18:0 (sn-1)	599.3 / 241.1	28.79	-65	12.38	negative

GL	lysoPI	lysoPI 18:1 (sn-1)	597.3 / 241.1	29.41	-65	12.55	negative
		lysoPI 18:2 (sn-1)	595.3 / 241.1	28.66	-65	9.00	negative
		lysoPI 18:3 (sn-1)	593.3 / 241.1	30.16	-65	11.44	negative
		lysoPI 19:0 (sn-1)	613.2 / 241.1	28.22	-65	5.94	negative
		lysoPI 20:0 (sn-1)	617.3 / 241.1	30.16	-65	5.46	negative
		lysoPI 20:1 (sn-1)	615.3 / 241.1	28.39	-65	ND	negative
		lysoPI 20:2 (sn-1)	613.3 / 241.1	28.49	-65	6.16	negative
		lysoPI 20:3 (sn-1)	611.3 / 241.1	28.66	-65	4.12	negative
		lysoPI 20:4 (sn-1)	609.3 / 241.1	27.91	-65	19.90	negative
		lysoPI 20:5 (sn-1)	607.3 / 241.1	29.16	-65	15.93	negative
		lysoPI 22:0 (sn-1)	655.4 / 241.1	29.79	-65	15.53	negative
		lysoPI 22:4 (sn-1)	647.3 / 241.1	28.91	-65	2.78	negative
		lysoPI 22:6 (sn-1)	643.3 / 241.1	28.49	-65	11.11	negative
	lysoPG	lysoPG14:0(sn-2)	455.3 / 227.0	19.53	-35	0.14	negative
		lysoPG15:0(sn-2)	469.3 / 227.0	12.9	-35	15.24	negative
		lysoPG15:1 (sn-1)	467.3 / 227.0	1.01	-35	4.78	negative
		lysoPG16:0(sn-2)	483.3 / 227.0	18.28	-35	2.05	negative
		lysoPG16:1(sn-1)	481.3 / 227.0	18.78	-35	6.49	negative
		lysoPG17:0(sn-1)	497.3 / 227.0	18.15	-35	3.76	negative
		lysoPG17:1(sn-1)	495.3 / 227.0	15.53	-35	11.94	negative
		lysoPG18:0(sn-1)	511.3 / 227.0	17.28	-35	4.94	negative
		lysoPG18:1(sn-1)	509.3 / 227.0	17.78	-35	0.74	negative
		lysoPG18:2(sn-1)	507.3 / 227.0	18.15	-35	11.94	negative
		lysoPG18:3(sn-1)	505.3 / 227.0	17.78	-35	11.59	negative
		lysoPG19:0(sn-1)	525.3 / 227.0	16.53	-35	11.92	negative
		lysoPG20:0(sn-1)	539.3 / 227.0	18.28	-35	7.02	negative
		lysoPG20:1(sn-1)	537.3 / 227.0	17.03	-35	6.77	negative
		lysoPG20:2(sn-1)	535.3 / 227.0	17.53	-35	18.03	negative
		lysoPG20:3(sn-1)	533.3 / 227.0	17.9	-35	12.35	negative
		lysoPG20:4(sn-1)	531.3 / 227.0	17.65	-35	6.04	negative
		lysoPG20:5(sn-1)	529.3 / 227.0	22.53	-35	9.54	negative
		lysoPG22:0(sn-1)	567.4 / 227.0	15.27	-35	9.13	negative

GL	lysoPS	lysoPG22:4(sn-1)	559.3 / 227.0	17.15	-35	0.82	negative
		lysoPG22:6(sn-1)	555.3 / 227.0	18.03	-35	12.26	negative
		lysoPS14:0(sn-2)	468.3 / 381.3	36.42	-25	5.03	negative
		lysoPS15:0(sn-2)	482.3 / 395.3	33.79	-25	0.74	negative
		lysoPS15:1(sn-1)	480.3 / 393.3	32.92	-25	4.56	negative
		lysoPS16:0(sn-2)	496.3 / 409.3	34.79	-25	1.25	negative
		lysoPS16:1(sn-1)	494.3 / 407.3	34.54	-25	1.34	negative
		lysoPS17:0(sn-1)	510.3 / 423.3	34.11	-25	11.70	negative
		lysoPS17:1(sn-1)	508.3 / 421.3	34.67	-25	7.31	negative
		lysoPS18:0(sn-1)	524.3 / 437.3	33.29	-25	3.93	negative
		lysoPS18:1(sn-1)	522.3 / 435.3	34.04	-25	2.97	negative
		lysoPS18:2(sn-1)	520.3 / 433.3	34.43	-25	5.19	negative
		lysoPS18:3(sn-1)	518.3 / 431.3	34.42	-25	4.85	negative
		lysoPS19:0(sn-1)	538.3 / 451.3	32.29	-25	2.67	negative
		lysoPS20:0(sn-1)	552.3 / 465.3	35.42	-25	14.11	negative
		lysoPS20:1(sn-1)	550.3 / 463.3	32.79	-25	1.64	negative
		lysoPS20:2(sn-1)	548.3 / 461.3	32.29	-25	0.86	negative
		lysoPS20:3(sn-1)	546.3 / 459.3	33.17	-25	4.00	negative
		lysoPS20:4(sn-1)	544.3 / 457.3	34.17	-25	5.02	negative
		lysoPS20:5(sn-1)	542.3 / 455.3	34.92	-25	5.04	negative
		lysoPS22:0(sn-1)	580.4 / 493.4	31.41	-25	0.02	negative
		lysoPS22:4(sn-1)	572.4 / 485.4	32.29	-25	0.14	negative
		lysoPS22:6(sn-1)	568.4 / 481.4	34.17	-25	10.78	negative
Ceramide	C1P	C1P120	560.6 / 78.9	26.66	-65	4.90	negative
		C1P160	616.7 / 78.9	41.67	-65	12.05	negative
		C1P240	728.9 / 78.9	37.92	-70	5.69	negative
		DHC1P160	562.6 / 78.9	26.16	-65	1.36	negative
		DHC1P240	730.9 / 78.9	15.53	-72	12.18	negative
Glycolipids	Sulfatide	SulfatideC120	722.5/96.9	6.47	-100	12.26	negative
		SulfatideC160	778.6 / 96.9	ND	-100	1.36	negative
		SulfatideC240	890.0 / 96.9	7.18	-100	5.13	negative

RT#: Retention Time, CE*: Collision Energy, RSD%: Relative Standard Deviation

Figure S1. PSL-DA model

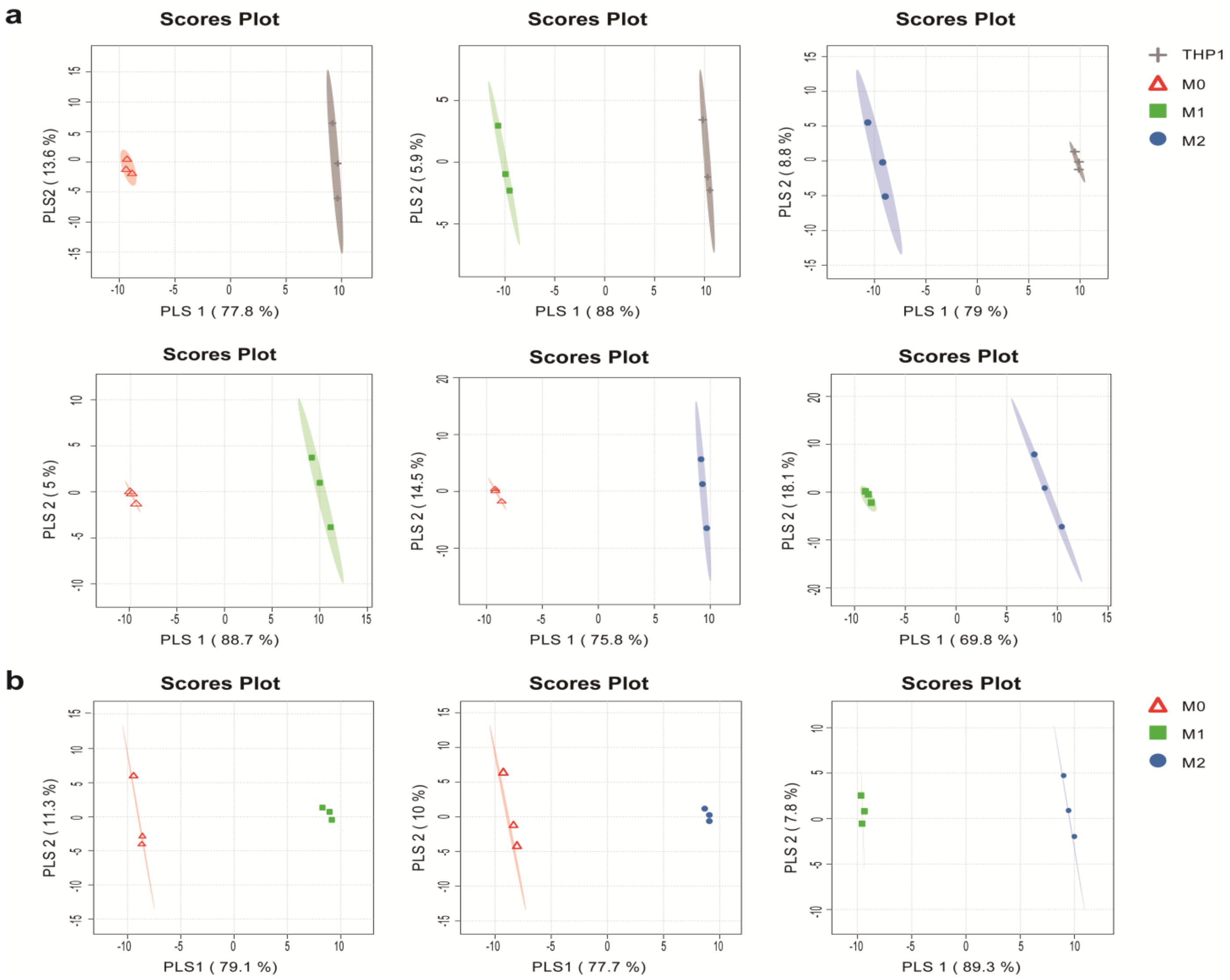

Figure S2

The changes of lipid species are observed during human primary monocyte derived macrophage differentiation and macrophage polarization

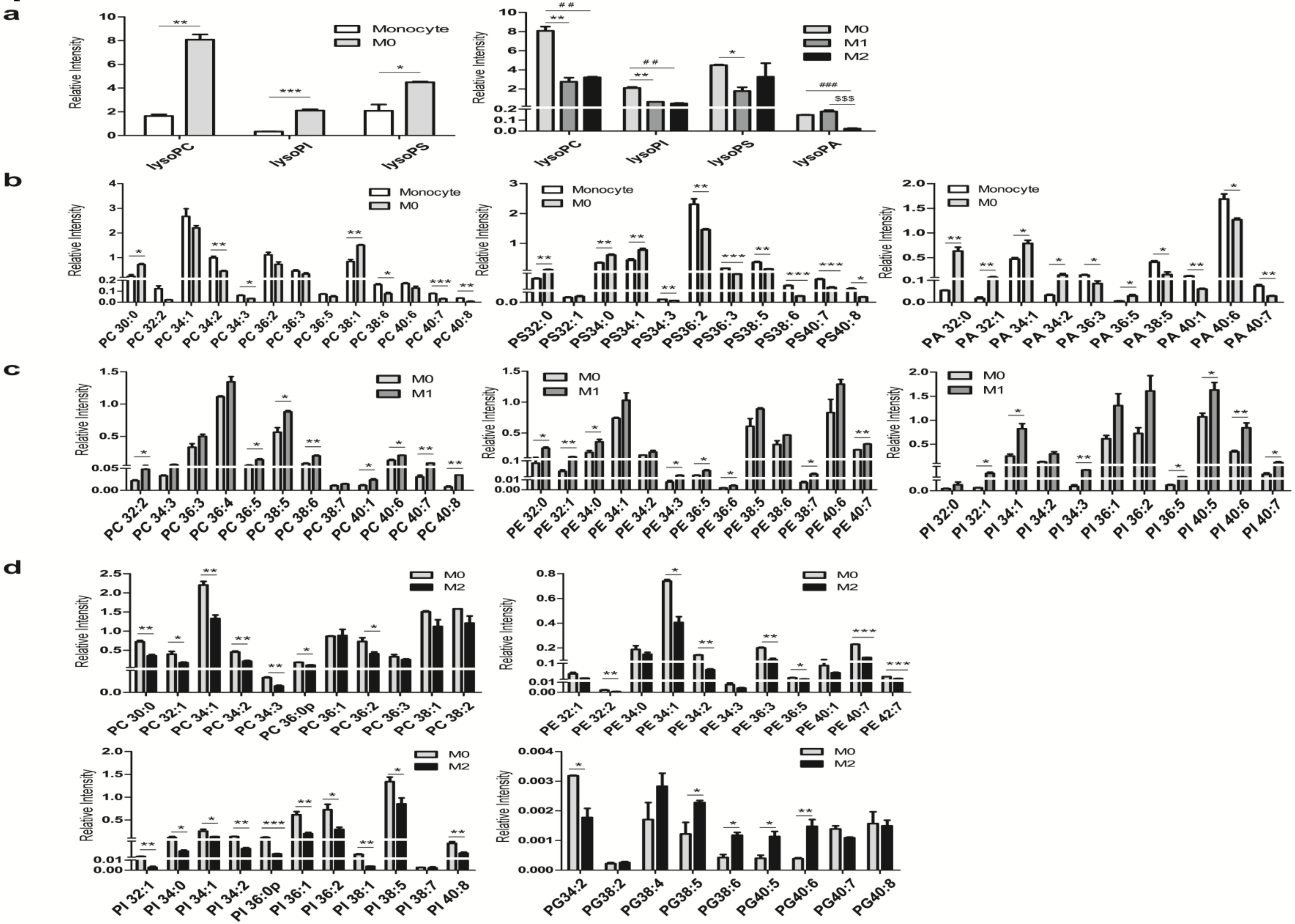

Figure S3

The changes of lysophospholipid molecular species are observed during THP-1 derived macrophage differentiation and macrophage polarization

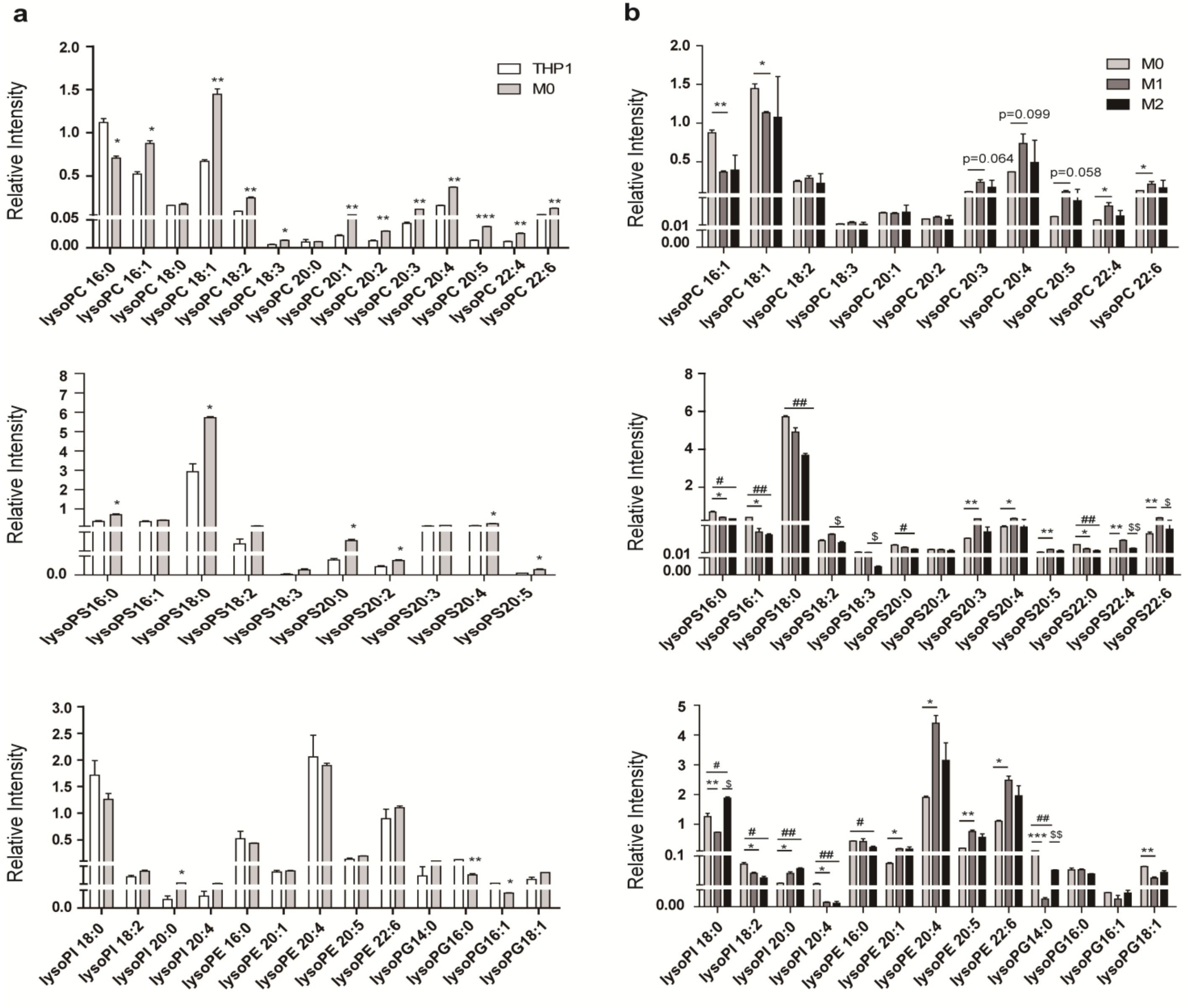

Figure S4

The variation of AA-containing lipid species during the courses of human monocyte-macrophage differentiation and macrophage polarization

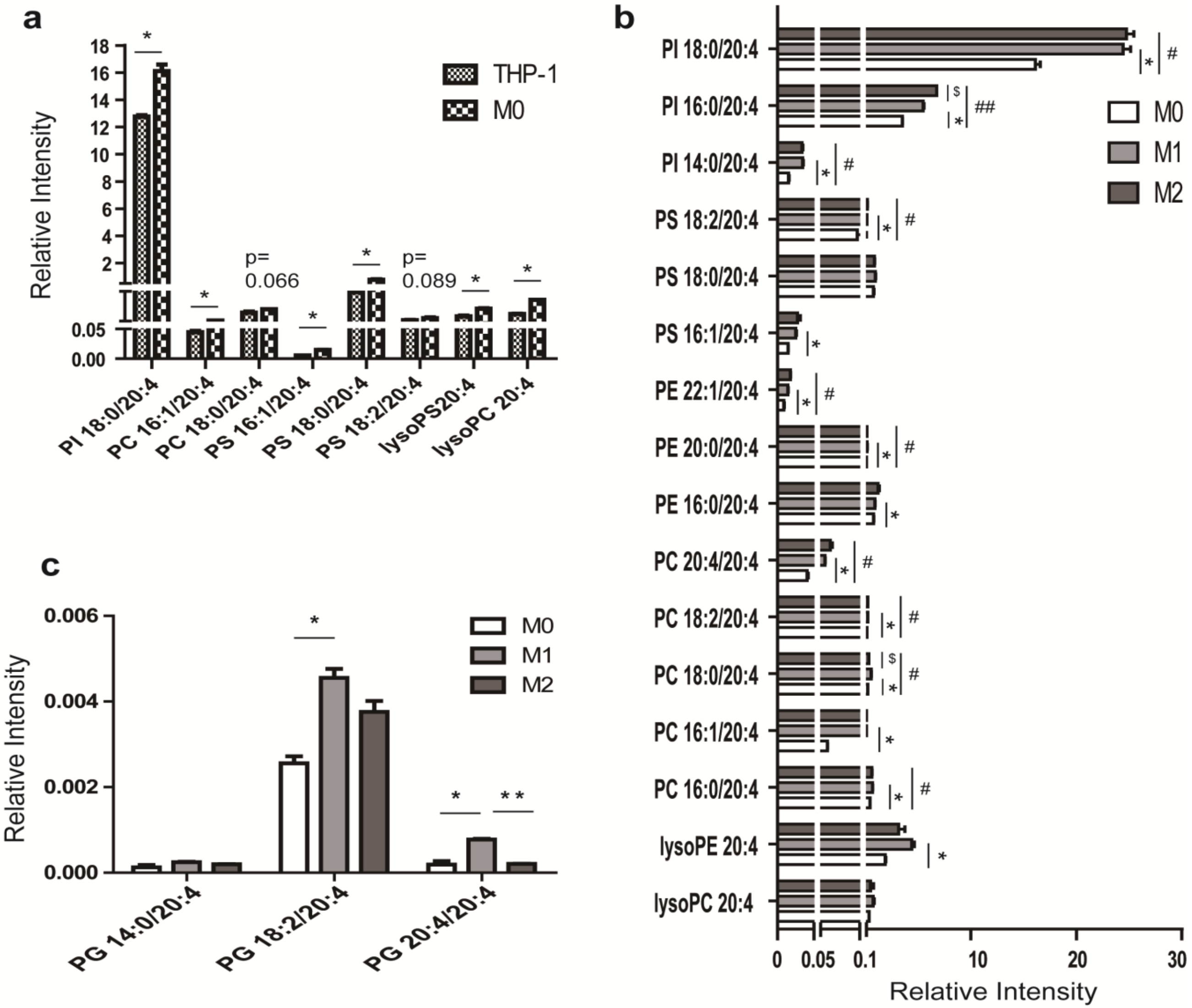

Figure S5 The Liquid chromatographic separation conditions

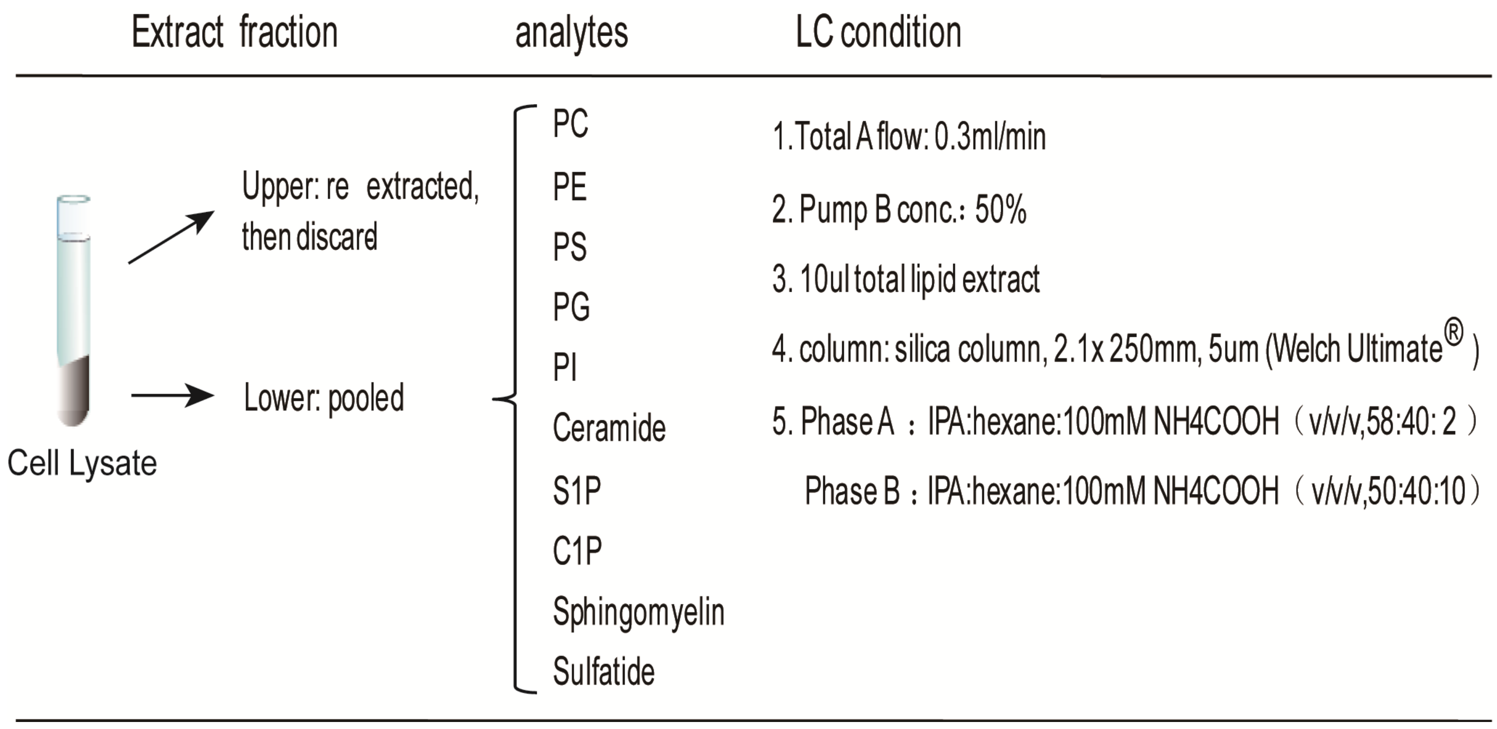

Figure S6

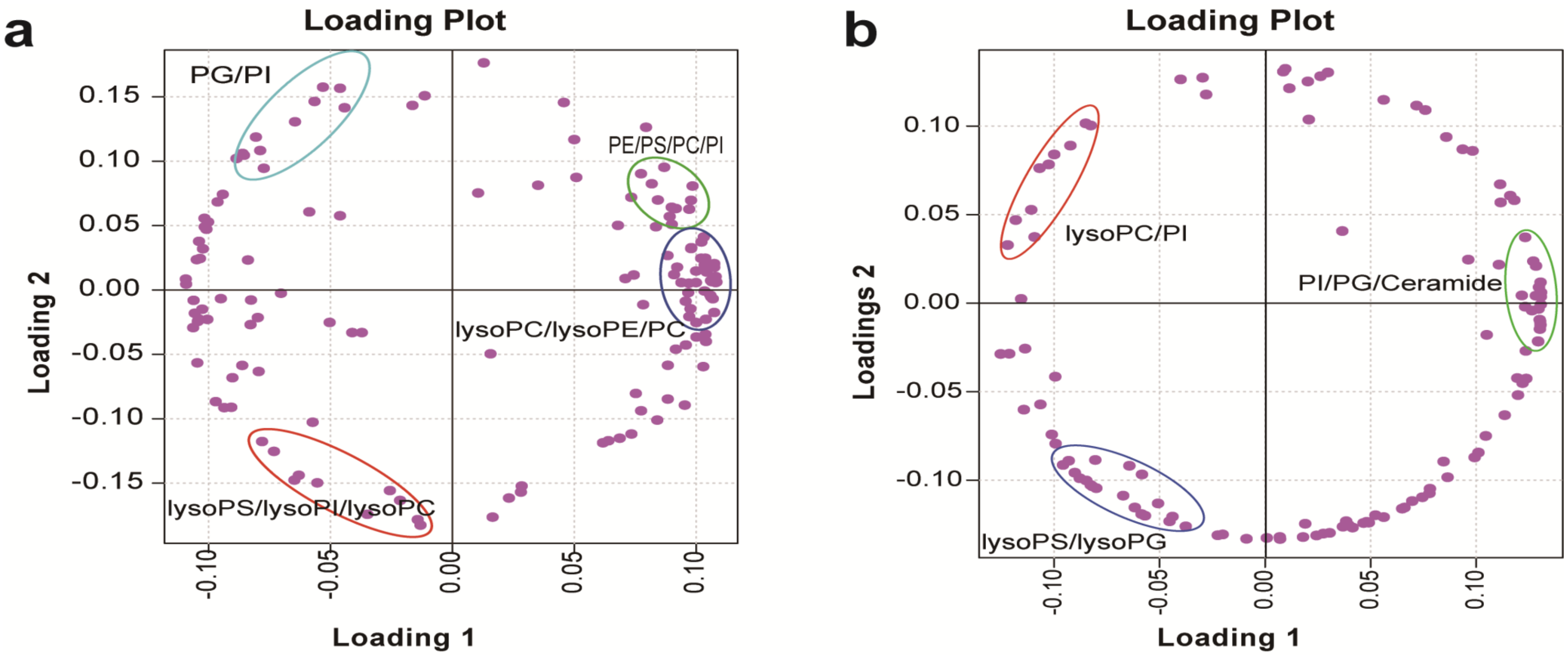
a
Loading Plot
PG/PI
PE/PS/PC/PI
lysoPC/lysoPE/PC
lysoPS/lysoPI/lysoPC
0.15
0.10
0.05
0.00
-0.05
-0.10
-0.15
-0.10
-0.05
0.00
0.05
0.10
Loading 2
Loading 1
b
Loading Plot
lysoPC/PI
PI/PG/Ceramide
lysoPS/lysoPG
0.10
0.05
0.00
-0.05
-0.10
-0.10
-0.05
0.00
0.05
0.10
Loadings 2
Loading 1

Table S2. List of primer sequences used in qRT-PCR experiments

Species	Genes	Primers	Sequences
human	CD11b	Forward	GTGAAGCCAATAACGCAGC
human	CD11b	Reverse	CTCCCATCCGTGATGACAAC
human	CD36	Forward	TGTAACCCAAGACGCTGAGG
human	CD36	Reverse	GAAGGTTCGAAGATGGCACC
human	CCL3	Forward	CAGCCAGGTGTCATTTTCCT
human	CCL3	Reverse	TCAGGCATTCAGTTCCAGGT
human	TNF-α	Forward	TAGCCCATGTTGTAGCAAACC
human	TNF-α	Reverse	ATGAGGTACAGGCCCTCTGAT
human	CCL17	Forward	ACTGCTCCAGGGATGCCATCGTTTTT
human	CCL17	Reverse	ACAAGGGGATGGGATCTCCCTCACTG
human	CCL22	Forward	TGATTACGATCCGTTACCGTCT
human	CCL22	Reverse	CCTGAAGGTTAGCAACACCAC
human	ACTIN	Forward	CCTAGAAGCATTTGCGGTGG
human	ACTIN	Reverse	GAGCTACGAGCTGCCTGACG
mouse	NOS2	Forward	AAACCCCTTGTGCTGTTCTC
mouse	NOS2	Reverse	ATACTGTGGACGGGTCGATG
mouse	CXCL10	Forward	AAGTGCTGCCGTCATTTTCT
mouse	CXCL10	Reverse	CCTATGGCCCTCATTCTCAC
mouse	Arg-1	Forward	GCAGAGGTCCAGAAGAATGG
mouse	Arg-1	Reverse	AGCATCCACCCAAATGACAC
mouse	Mrc-1	Forward	GGAGGCTGATTACGAGCAGT
mouse	Mrc-1	Reverse	CATAGGAAACGGGAGAACCA
mouse	ACTIN	Forward	GTGCTATGTTGCTCTAGACTTCG
mouse	ACTIN	Reverse	ATGCCACAGGATTCCATACC

Table S3. Comparison of lipidomic characteristics among human THP-1, human PBMC and mouse RAW264.7 macrophage

Differentiation/Polarization process	Human THP-1	Human PBMC	Mouse RAW264.7
Monocyte to M0	lysoPC and lysoPS ↑	lysoPC,lysoPS,lysoPI ↑<br>PUFA to MUFA (PC, PS, PA species)	
M0 to M1	PE,PG ↑<br>lysoPI ↓ lysoPE ↑<br>MUFA to PUMA(PS,PC,PI species)<br>Fatty acid lengthen(PS,PC,PI species)	PC,PE,PI ↑<br>lysoPC, lysoPS, lysoPI ↓	PG, PC, PS↑<br>lysoPG ↑, lysoPC ↓
M0 to M2	PE,PG↑<br>lysoPI ↑<br>MUFA to PUMA (PS,PC,PI species)<br>Fatty acid lengthen(PS,PC,PI species)	PC,PE,PI ↓ ,PG↑<br>lysoPC, lysoPI, lysoPA ↓	PS, PE, PC ↑<br>lysoPS, lysoPG, lysoPI ↑

"↑": the intensity of lipid was increased

" ↓ ":the intensity of lipid was decreased

Figure S1. PSL-DA model
(a) Human macrophage PLS-DA model.
(b) Mouse macrophage PLS-DA model.

Figure S2
The changes of lipid species are observed during human primary monocyte derived macrophage differentiation and macrophage polarization
(a) In the resting macrophages, the intensity of lysoPC, lysoPS and lyoPI was found to be significantly elevated. The intensity of lysoPA was significantly decreased in M2 macrophage.
(b) The data shown a decrease of PUFA accompanied with an increase of MUFA and the saturated species, especially the PC, PS and PA species, in human primary monocyte-macrophage differentiation.
(c) An increase of PC, PE and PI species were found in human primary monocyte derived M1 macrophage.
(d) When compared with M1 cells, there seemed to be an opposite tendency for M2 cells to exhibit decreases in PC, PE and PI species and increases in PG species.

Figure S3
The changes of lysophospholipid molecular species are observed during THP-1 derived macrophage differentiation and macrophage polarization
(a) Mono- and poly- unsaturated lysoPC species are significantly elevated in resting macrophages when compared with THP-1.
(b) In the course of macrophage polarization, long-chain polyunsaturated lipids are increased in the LPS stimulation of macrophages, especially lysoPC, lysoPS and lysoPE species. It seemed to be an opposite tendency for the LPS- and IL-4-treated cells to exhibit decreases in polyunsaturated lysoPI (C18:2, C20:4) and increases in saturated lysoPI (*$p<0.05$, **$p<0.01$, ***$p<0.001$.* represent comparison between M1 and M0, # represent comparison between M2 and M0, $ represent comparison M1 and M2).

Figure S4
The variation of AA-containing lipid species during the courses of human monocyte-macrophage differentiation and macrophage polarization
(a) AA-containing glycerophospholipids were elevated in PMA-induced macrophages.
(b) Almost all the AA-containing species we detected were increased in M1 and M2 macrophages when compared with M0 macrophages.

(* represent comparison between M1 and M0, # represent comparison between M2 and M0, $ represent comparison M1 and M2)
(c) PG40:8(C20:4/20:4) is the top of lipid elevated in M1 macrophage compared with M0 and M2 cells.

Figure S5 The Liquid chromatographic separation conditions

Figure S6 The loadings plots that correspond to the score plots that are shown in figures 2d and 4d
(a) The loading plot is correspond to the score plot presented in figure 2d. The lipid peaks that labeled “PG/PI” in the second quadrant are responsible for separating THP-1 monocyte population. The lipid peaks that labeled “lysoPS/lysoPI/lysoPC” in the third quadrant are responsible for separating human M0 macrophage population. Similarly, the lipid peaks that labeled “PE/PS/PC/PI” and “lysoPC/lysoPE/PC” are responsible for separating human M1 and M2 macrophage population respectively.
(b) The loading plot is correspond to the score plot presented in figure 4d. The lipid peaks that labeled “;lysoPC/PI” in the second quadrant are responsible for separating mouse M0 macrophage population. The lipid peaks that labeled “PI/PG/Ceramide” are responsible for separating mouse M1 macrophage population. Similarly, the lipid peaks that labeled “lysoPS/lysoPG” in the third quadrant are responsible for separating mouse M2 macrophage population.
